# A comparison of the accuracy of iTRAQ quantification by nLC-ESI MSMS and nLC-MALDI MSMS methods

**DOI:** 10.1016/j.jprot.2010.03.003

**Published:** 2010-05-07

**Authors:** Sally L. Shirran, Catherine H. Botting

**Affiliations:** Centre for Biomolecular Sciences, Biomolecular Sciences Building, North Haugh, University of St Andrews, Fife, KY16 9ST, United Kingdom

**Keywords:** iTRAQ quantification, nLC-ESI MSMS, nLC-MALDI MSMS, Mascot, ProteinPilot

## Abstract

The accuracy of quantification obtained using the iTRAQ labelling methodology for measuring protein ratios more extreme than 1:1 was investigated. A comparison of nLC-ESI MSMS and nLC-MALDI MSMS analysis routes was performed. A fixed concentration of a standard six protein mix was spiked with two proteins at a range of concentrations. The two data analysis programmes, Mascot and ProteinPilot Paragon, were also compared. Whilst the lower ratios could be measured accurately, greater discrepancies were seen for the higher ratios, particularly by nLC-ESI MSMS. Filtering out the weaker reporter ion signals improved the accuracy of the ratios: this is likely due to several factors which are explored in more detail. Overall, analysis by nLC-MALDI MSMS followed by Mascot interpretation gave the most accurate results.

## Graphical abstract

**Figure:**
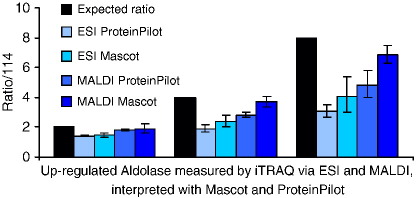

## 1 Introduction

A main objective of proteomics research is to systematically identify and quantify the proteins in a given proteome [1]. The iTRAQ method of quantitative proteomics [2] is one of the leading quantitative techniques and has been demonstrated in many organisms and tissues [3–5]. The method involves labelling a sample from each condition/time point, normally at the peptide level (although protein-labelling is also possible), with one of a range of four or eight isotopically labelled reagents. These reagents contain a balance and reporter moiety giving isobaric labels at the MS level, whilst fragmentation gives reporter ions under MSMS conditions. The reporter ion peaks are then quantified.

The analysis of iTRAQ-labelled samples from complex mixtures commonly requires nLC-MSMS, with either ESI or MALDI as the ionization method. In nLC-ESI MSMS the chromatographic separation is coupled directly to the MS analysis. Peptides are detected, selected and fragmented in real time, as the column eluent sprays into the instrument. Particularly in older instruments, MS and MSMS scan times are preset at the beginning of the run as a compromise between signal intensity and the requirement to analyse as many peptides as possible in a chromatographic window. In contrast, during nLC-MALDI MSMS [6], the nanoflowLC column eluent is collected as droplet-sized fractions on the MALDI target plate by a spotting robot, which also adds matrix to the spots. nLC-MALDI MSMS has the advantage that the collection of MSMS data is decoupled from the LC. Typically, after a MS survey scan, the ion chromatogram is generated in silico and the peptides selected for MSMS at their maxima. This minimises the collection of redundant data and ensures the collection of isobaric peptides eluting at similar time points but with different elution profiles.

nLC-MALDI MSMS has the further advantage, as in traditional MALDI, that it is more tolerant to salts and interfering compounds in the sample or eluents, allowing a wider range of chromatographic conditions to be utilised. Additionally there is no time constraint on analysing a particular fraction, allowing mining down to obtain MSMS, in practise, until the sample has been ablated. Furthermore, the sample is stored on the plate and can be archived for later analysis [7]. Nevertheless, the nature of the large-scale nLC-MALDI MSMS experiment is time consuming precisely because the analysis is decoupled, with the LC and MS performed sequentially, typically with manual intervention in between.

As MALDI and ESI ionization give, to some extent, complementary results [8,9], it may be of benefit to use both analysis techniques in parallel to achieve maximum proteome coverage. However, are both analysis routes equally effective for quantitative analysis performed using iTRAQ labelling?

Whilst earlier literature reports show that low ratios of up or down-regulation (1:1 or 1:2) can be measured accurately by both ESI and MALDI routes [6,10,11] some laboratories are reporting an underestimation of the degree of up- and down-regulation when using iTRAQ quantification to look at larger changes. This is particularly evidenced in the ABRF Proteomics Research Group 2007 Advanced Proteomics Study [12] where standard proteins, spiked into an E. coli cell lysate background, at ratios of 1:4.6 and 1:10 were significantly underestimated by iTRAQ analysis. Furthermore, the iTRAQ ratios reported by different laboratories all showed similar deviation from the expected value, suggesting a common underlying problem. Likewise, DeSouza et al. [13] noted that the ratio for the relative abundance of pyruvate kinase measured in a discovery-phase study of endometrial tissue performed using iTRAQ was lower, at 2, compared to a ratio of 4 obtained with the mTRAQ variant of iTRAQ, used for absolute quantification [14], when analysed on a Q-Trap instrument. Patel et al. [15] compared the label free MS^E^ quantification method with the iTRAQ method, using a Q-ToF instrument, and saw discrepancies in the degree of up-regulation of up to a factor of 9.2 between the two quantification methods, with the iTRAQ method giving lower ratios. Pierce et al. [16], investigating the 8plex iTRAQ reagents, with analysis on a QStar XL instrument, saw underestimation of the degree of up and down-regulation, in a defined mixture, by up to a factor of 5 (a 1:10 ratio measuring only 1:2). However, these results contrast with the work of Kuzyk et al. [7] who have recently shown 10:1 ratios that were only underestimated by 14% (QStar Elite) and 20% (4800 MALDI TOF/TOF Analyser) and of Ow et al. [17] who measured ratios up to 1:10 accurately using 8plex reagents.

Here we describe the analysis of an iTRAQ labelled sample by nLC-ESI MSMS, on a QStar XL, and by nLC-MALDI MSMS, on a 4800 MALDI TOF/TOF Analyser, with the aim of investigating the accuracy and precision of the two data sets. The sample was created by spiking known ratios of two proteins into a constant background of a further six proteins at a 2, 4, and 8 fold excess, and a 2, 4 and 8 fold reduction. The two data sets were each analysed using the software packages Mascot and ProteinPilot Paragon and the quantification results compared. These software packages are two of a number of packages that support iTRAQ data analysis: Mascot (MatrixScience), ProteinPilot Paragon and its forerunner ProQuant (Applied Biosystems), SpectrumMill (Agilent), Warp LC (Bruker), Scaffold Q+ [18] (Proteome Software Inc) and Peaks [19] (Bioinformatics Solutions Inc) amongst others. The packages differ in the way that the reporter ion peak intensity is calculated and the statistical methods that are applied to the data.

## 2 Materials and methods

### 2.1 Preparation of 8 protein sample

A tryptic digest of a six protein mix (Dionex, Sunnyvale, CA) which had been reduced and alkylated, with iodoacetic acid, was spiked with varying amounts of trypsin digested rabbit muscle aldolase (Ald) and human carbonic anhydrase (CAH) (Sigma-Aldrich, Poole, UK) in 100 mM triethylammonium bicarbonate (TEAB). The spike proteins were not reduced and alkylated. The four samples to be labelled with the 4plex iTRAQ reagents (Applied Biosytems, Foster City, CA) 114, 115, 116 and 117 respectively, each contained 5 pmol of each of the six proteins in the mix (lysozyme C (LysoC), cytochrome C (CytoC), ß-galactosidase (βGal), bovine serum albumin (BSA), alcohol dehydrogenase (ADH) and serotransferrin (TF)). CAH and Ald peptides were added as follows: iTRAQ label 114: 20 pmol CAH, 2.5 pmol Ald; iTRAQ label 115: 10 pmol CAH, 5 pmol Ald; iTRAQ label 116: 5 pmol CAH, 10 pmol Ald and iTRAQ label 117: 2.5 pmol CAH, 20 pmol Ald. Hence the combined labelled samples contained 195 pmol of digested protein. This provided a sample which mimicked CAH down-regulation by 2, 4 and 8 fold and Ald up-regulation by 2, 4 and 8 fold relative to the 114 label.

The peptide mixtures were concentrated to dryness and resuspended in TEAB (1.5 μL, 0.5M). The appropriate iTRAQ reagent (3.5 μL, 0.05 unit, from a tube contents (1 unit) resuspended in 70 μL ethanol) was added to each sample and the individual reactions incubated at room temperature for 1 h. The four labelling reactions were then combined and the sample concentrated to dryness. The mixture was resuspended in cation exchange load buffer (10 mM K_2_HPO_4_, pH 3.0, 25% ACN) and loaded on to a cation exchange cartridge (as shipped with iTRAQ Reagent Methods Development Kit, Applied Biosystems), which had been pre-equilibrated with load buffer. After washing away non-binding contaminants with load buffer, the peptides were eluted with 500 μL, 10 mM K_2_HPO_4_, pH 3.0, 320 mM KCl, 25% ACN. An equal volume of 0.2% TFA was added to the eluent and it was desalted using a Sep-Pak 100mg C18 cartridge (Waters, Elstree, UK). The eluent was concentrated to dryness and resuspended in 0.5% formic acid. Half of the sample (97.5 pmol) was analysed by nLC-ESI MSMS and half (97.5 pmol) by nLC-MALDI MSMS. The experiment was performed in triplicate, using different batches of peptide and iTRAQ reagents.

### 2.2 Sample analysis by nLC-MALDI MSMS

The peptides were separated using a Dionex UltiMate 3000 nanoLC equipped with a PepMap100 C18 300 μm × 5 mm trap and 75 μm × 15 cm column (Dionex), using a 3.5 h gradient of increasing acetonitrile concentration, containing 0.05% TFA (5-35% ACN in 3 h, 35–50% in a further 30 min, followed by 95% ACN to clean the column). The eluent was spotted onto a MALDI target plate, along with α-cyano-4-hydroxycinnamic acid (2 mg/mL in 70% ACN, 0.1% TFA) at 0.98 μL/min, using a Dionex Probot spotter, collecting 20 s fractions.

The nLC-MALDI fractions were analysed using an Applied Biosystems 4800 MALDI TOF/TOF Analyser equipped with a Nd:YAG 355 nm laser in a plate-wide data dependent manner. All spots were initially analysed in positive MS mode over the range 800 to 4000 *m*/*z* by averaging 1000 laser spots. The MS ions that satisfied the precursor criteria (200 ppm fraction to fraction precursor exclusion, S/N ratio > 20) were selected for subsequent MSMS, from the spot where the MS ion gave the highest counts, with up to 5 MSMS being acquired from each spot, selecting the strongest precursor ion first. MSMS spectra were acquired to a maximum of 3000 laser shots or until the accumulated spectrum reached a S/N ratio of 35 for 10 peaks. All MSMS data were acquired using 1 keV collision energy.

### 2.3 Sample analysis by nLC-ESI MSMS

The peptides were separated using a Dionex UltiMate nanoLC equipped with a PepMap100 C18 300 μm × 5 mm trap and 75 μm × 15 cm column (Dionex), using a 3.5 h gradient of increasing ACN concentration, containing 0.1% formic acid (5–35% ACN in 3 h, 35–50% in a further 30 min, followed by 95% ACN to clean the column). The eluent was sprayed into an Applied Biosystems QStar XL tandem mass spectrometer and analysed in Information Dependent Acquisition (IDA) mode using Analyst QS 1.1 software. The MS was collected for 1 s, and the two strongest 2+ or 3+ ions that met the MS criteria (ions greater than 300 *m*/*z* but less than 1200 *m*/*z* that exceed 10 counts, mass tolerance 100 ppm) were selected for 3 s MSMS each. These masses were then excluded from analysis for the next 60 s. A rolling collision energy was employed for fragmentation, set 10 V higher than that normally used for peptides, to provide sufficient peptide fragmentation and generation of the iTRAQ reporter groups.

### 2.4 Data analysis

The data files were processed by both ProteinPilot 2.0 (Applied Biosystems) using the Paragon algorithm [20] and Mascot v2.2 [21] (Matrix Science, London, UK). All searches were performed against the UniProt database (Swiss-Prot and Trembl databases combined, 9 April, 2009, 7,966,092 sequences). Automatic isotope correction was carried out by both software packages using the values supplied with the Applied Biosystems reagents. For ProteinPilot Paragon, iodoacetic acid was selected as the cysteine modification agent, trypsin as the digestion enzyme, ‘biological modifications’ were selected as the ‘ID focus’ and a ‘Thorough ID Search Effort’ was selected. Although the protein set was too small to expect truly meaningful values, the Proteomics System Performance Evaluation Pipeline Software (PSPEP) using reversed protein sequences was used to calculate the number of false positive proteins expected at a 95% confidence level. For Mascot, the nLC-ESI MSMS data for doubly and triply charged precursor ions were converted to centroid data, without smoothing, using the Analyst QS1.1 mascot .dll data import filter. The ‘MS/MS averaging of IDA dependents’ had a precursor mass tolerance for grouping of 0.1 and the maximum number of cycles between groups and minimum number of cycles per group were both set to 1. The MS/MS settings include: spectra de-isotoped — except for the iTRAQ reporter region, peak areas reported, spectra rejected if they contained less than 10 peaks, and peaks not removed if they were close to the precursor *m*/*z*. The nLC-ESI MSMS data were searched with a tolerance of 0.08 Da for the precursor ions and 0.2 Da for the fragment ions. The nLC-MALDI MSMS data were extracted using TS2Mascot 1.0.0 (Matrix Science) and the data saved to a peak list. The nLC-MALDI MSMS data were searched with tolerances of 100 ppm for the precursor ion and 0.5 Da for the fragment ions. For both ionization routes the following settings were used: trypsin was the cleavage enzyme, one missed cleavage, carboxymethyl modification of cysteines was a fixed modification and methionine oxidation was selected as a variable modification. The following settings were used to manipulate the quantification results: the protein ratio type was the ‘weighted’ geometric mean, there was no normalisation, outlier removal was ‘automatic’ (Dixon's method up to 25 data points, Rosner's method above 25 data points), the peptide threshold was ‘at least homology’ (peptide score does not exceed absolute threshold but is an outlier from the quasi-normal distribution of random scores), the minimum number of peptides was two and peptides were required to be the top ranking peptide matches (‘red’). An automatic decoy database search was also performed.

## 3 Results and discussion

### 3.1 Global comparison of nLC-ESI MSMS and nLC-MALDI MSMS analysis

**Fig. 1:**
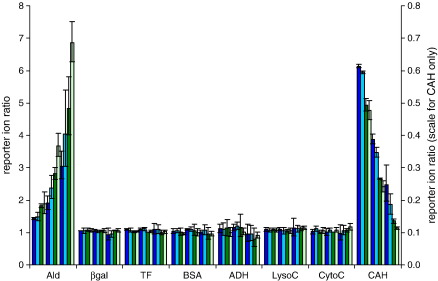
Comparison of iTRAQ quantification analysis for the 8 protein sample. Each protein has 12 ratios plotted, the first 4 show the 115:114 ratio, the next 4 the 116:114 ratio and the last 4 the 117:114 ratio. Blue indicates nLC-ESI MSMS, green indicates nLC-MALDI MSMS. Dark shades are ProteinPilot Paragon analysis, light shades Mascot analysis. Hence**, dark blue**: nLC-ESI MSMS and ProteinPilot Paragon, **light blue**: nLC-ESI MSMS and Mascot, **dark green**: nLC-MALDI MSMS and ProteinPilot Paragon, **light green**: nLC-MALDI MSMS and Mascot. Values plotted are geometric mean ratios of the 3 software derived ratios from the triplicate runs with the 95% confidence level for the geometric standard deviations shown by error bars. CAH, the down-regulated protein, is plotted on the secondary axis on the right.

The trypsin digest of an eight protein mix labelled with iTRAQ reagents was analysed by both nLC-ESI MSMS (QStar XL) and nLC-MALDI MSMS (4800 MALDI TOF/TOF Analyser) methods and the data analysed using Mascot and ProteinPilot Paragon with the settings described above. The results were compared. Both techniques provided confident identifications of the expected proteins with high peptide coverage (Table 1). The six background proteins from a pre-digested commercial protein mix were spiked with digested peptides from Ald (up-regulation by a factor of 2, 4 and 8) and CAH (down-regulation by a factor of 2, 4 and 8). The spike proteins were medium sized proteins which should generate enough peptides to give statistically significant results. The concentration of the six background proteins remained constant between each labelling experiment and, therefore, values of 1 were expected for the 115:114, 116:114 and 117:114 reporter ion ratios. Fig. 1 shows ratios close to 1 were achieved in each of the triplicate experiments, using both analysis methods and analysis software packages. The geometric mean of the geometric standard deviations between the triplicate measurements was 10% at the 95% confidence interval for the six background proteins and for the Ald and CAH measurements, indicating reproducible experimental technique. However the accuracy of the results for those proteins showing changes in ‘expression’ level was low, with the degree of up-regulation of Ald, spiked in at 2, 4 and 8 fold excess compared to the 114 labelled experiment, underestimated by both nLC-MALDI MSMS and nLC-ESI MSMS, but more so by the latter. Furthermore, greater underestimation was seen using ProteinPilot Paragon as the analysis software, compared to the results achieved with Mascot. Larger deviations from the true value were seen at higher degrees of up- or down-regulation, particularly when using nLC-ESI MSMS analysis, with discrepancies as high as 62%. Similarly the degree of down-regulation of CAH was also underestimated by nLC-ESI MSMS (Fig. 1, right hand scale). The QStar XL and 4800 MALDI TOF/TOF Analyser data sets were analysed in more detail in order to determine the source(s) of these discrepancies.

### 3.2 Raw data assessment – reporter ions - nLC-MALDI MSMS of Ald

**Fig. 2:**
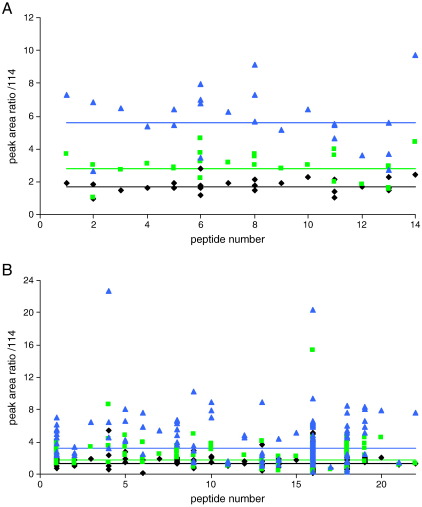
Reporter ion peak area ratios for Ald peptides. **A** nLC-MALDI MSMS analysis: reporter ion peak area ratios from triplicate analyses, calculated using peak areas from Data Explorer software, and isotope corrected, plotted for each Ald peptide identified with an above ‘homology threshold’ Mascot Ion Score. **B** nLC-ESI MSMS analysis: reporter ion peak area ratios from the triplicate analyses, calculated using peak areas from Analyst software, and isotope corrected, for each Ald peptide identified with an above ‘homology threshold’ Mascot Ion Score, a TIC above 12 for the most intense peak in the spectrum and a complete set of measureable reporter ions. **Black diamonds**: 115:114, **green squares**: 116:114 and **blue triangles**: 117:114. The **black**, **green** and **blue** lines indicate geometric mean for the 115:114, 116:114 and 117:114 data sets respectively. Expected ratios are 2, 4 and 8. Peptides are numbered in order of increasing *m*/*z*.

For the triplicate nLC-MALDI MSMS experiments, a total of 14 different peptide masses (between 5 and 10 per analysis and never more than two datasets for the same peptide mass) were used by Mascot for quantification of Ald, once those peptides with scores below the homology threshold, or with iTRAQ tyrosine modification, had been eliminated. (The reaction of the iTRAQ label with tyrosine is slower than that with amines and hence may not go to completion, which could lead to inaccurate ratios if these matches were included [2].) The reporter ion ratios relative to 114 were recalculated manually from the raw data, using Applied Biosystems Data Explorer software to calculate the MALDI peak areas, corrected for isotope impurities using the values from the Certificate of Analysis supplied with the reagents, and the reporter ion ratios calculated (Table 1 Supplementary Material). The ratios are plotted for each Ald peptide in Fig. 2A and the geometric mean and geometric standard deviation given in Table 2. It can be seen that there is a tendency for the raw data from peptides selected using the Mascot ‘at least homology’ threshold to give lower than expected peak area ratios.

**Fig. 3:**
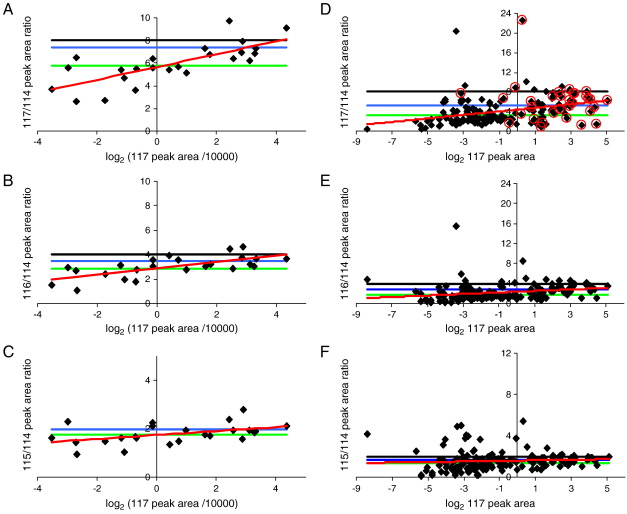
Plotting log_2_ 117 peak area against peak area ratio shows measured ratio tends towards expected ratio at greater 117 peak area. **A–C** nLC-MALDI MSMS analysis: reporter ion peak area ratios from the triplicate analyses, calculated using peak areas from Data Explorer software, and isotope corrected, for each Ald peptide with an above ‘homology threshold’ Mascot Ion Score, plotted against log_2_ (117 peak area/10,000) as a measure of reporter ion signal intensity. **D–F** nLC-ESI MSMS analysis: reporter ion peak area ratios from the triplicate analyses, calculated using peak areas from Analyst software, and isotope corrected, for each Ald peptide with an above ‘homology threshold’ Mascot Ion Score, a TIC above 12 for the most intense peak in the spectrum and a complete set of measureable reporter ions, plotted against log_2_ (117 peak area) as a measure of reporter ion signal intensity. **Black line** indicates expected ratio, **green line** indicates geometric mean, **blue** line indicates geometric mean after low intensity (ESI = 4, MALDI = 20,000) points are removed, **red line** is line of best fit. Points highlighted by red circles in **D** are data points collected at the peak (top 50%) of the eluting peptide chromatogram.

The peak area ratios 117:114, 116:114 and 115:114 were plotted against log_2_ of the peak area/10,000 of the 117 reporter ion as a measure of reporter ion intensity (Fig. 3A-C). The 117 reporter ion was chosen as the most intense and, therefore, most accurately measureable of the reporter ion peaks. The red lines of best fit are an indicator that the deviation from the expected peak area ratio is less at higher 117 peak areas. It would, therefore, be advantageous to remove data points below a certain 117 peak area threshold. If a threshold of 20,000 (1 on the graph log scale) is arbitrarily chosen the geometric mean values are adjusted as shown in Table 2 and by the blue lines on Fig. 3A-C). However, it should be noted that, with this threshold, the quantification now only uses the few most intense peaks for that protein from each run. Hu et al. [22] saw a similar effect when optimising proteomic analysis of mouse cerebellar dysfunction using a MALDI TOF/TOF 4700 Proteomics Analyzer, and also concluded that more accurate peptide expression ratios were obtained if the lower intensity peaks were filtered out.

### 3.3 Raw data assessment – reporter ions – nLC-ESI MSMS of Ald

Similarly, for the nLC-ESI MSMS data, reporter ion peak areas from each cycle (MSMS acquisition) selected by Mascot using the ‘at least homology’ threshold, that had a total ion count for the most intense peak in the spectrum of 12 or above and a complete set of measureable reporter ion peak areas were calculated using Applied Biosystems Analyst software. These values were corrected for isotope impurities and the reporter ion peak area ratios calculated (Table 2 Supplementary Material). The peak area ratios were plotted for each peptide (Fig. 2B). In contrast to the nLC-MALDI MSMS data, there tend to be more data points for many of the peptides. The geometric mean for the merged triplicate nLC-ESI MSMS data points is given in Table 2.

The peak area ratios 117:114, 116:114 and 115:114 were again plotted against log_2_ of the peak area of the 117 reporter ion (Fig. 3D-F). As shown by the red line of best fit, there is, once more, a tendency for the weaker data points to give less accurate, lower ratios. By removing data points below an arbitrary 117 peak area threshold of 4 (2 in log_2_), the accuracy of the peak area ratios can be improved as shown in Table 2 and as the blue line on the graphs. Therefore, removing those data points with the weakest 117 peak areas improves the accuracy of the ratios. However, the ratios are still underestimated, particularly, at the higher degrees of up- or down-regulation.

In this experiment all ratios are calculated relative to 114, which for Ald is the weakest peak and therefore possibly the least accurate in the reporter ion quadruplet. Ow et al. [17] showed that poor S/N led to increased errors when studying a 4 protein mix. Indeed we see for our data that the geometric mean of the ratio between each adjacent pair of reporter ions, which would be expected to be 2, is 1.36 (geo. SD: 1.71) for 116:115 and 1.80 (geo. SD: 1.78) for 117:116, compared to 1.35 (geo. SD: 1.84) for 115:114. So the 117:116 ratio is only 10% lower than expected compared to 33% lower, for the 115:114 and 116:115 ratios. Indeed the two outlying data points with 117/114 peak area ratios above 20 (see Fig. 3D and Supplementary Table 2) have anomalously small 114 peaks, hence the ratios relative to 114 are distorted although those between 115, 116 and 117 are reasonably accurate.

In this study MSMS was collected for 3 s regardless of signal intensity. However, Analyst QS2.0 software, for the newer QStar Elite instrument, allows collection of MSMS data until a predefined S/N is achieved, allowing shorter acquisition times for more intense ions, leading to the collection of more MSMS spectra in a given time period, when proteome coverage is important. However, for iTRAQ analysis, this S/N threshold can be increased to improve the signal intensity of data to be used for quantification. It may be this improvement, combined with other changes to the acquisition method discussed below, that led to the improved accuracy of the ratios reported by Kuzyk et al. [7] using a QStar Elite, over the results presented here using a QStar XL.

An alternative way of filtering the data would be to use only the reporter ion ratios acquired whilst a chromatographic peak is above 50% of its maximum intensity. This has some parallels to the MALDI acquisition, where MSMS is performed on the spot which represents the top of the chromatographic peak. In the combined triplicate data set 32 data points fulfil this criterion. They are depicted with red circles on Fig. 3D. Twenty one of these data points are above the arbitrary 117 peak area threshold cut-off value of 4 and eleven below. However, nine of the peaks above the threshold of 4 are not from above half height on the chromatographic peak. The geometric mean for the 32 data points is given in Table 2. As shown in Table 2, taking only the 21 data points that fulfil both criteria gives the most accurate set of ratios.

An alternative to removing the weaker peaks during the data manipulation stage would be to increase the number of counts required to trigger the collection of MSMS. However this would compromise the identification part of the analysis, so it is preferable to acquire as complete a data set as possible and then use data filtering tools to allow a data set that gives reliable quantification to be extracted.

Certainly a significant proportion of the poor nLC-ESI MSMS data is from analysis of material from chromatographic tailing. This is occurring, in part, due to the relative lack of complexity of the eight protein sample, compared to a ‘real’ sample, which promotes reanalysis of the chromatographic tail, as no ‘new’ peptides are eluting and also due to the high concentration of Ald peptides analysed (18.75 pmol per instrument run). This is excessive for identification, as the QStar XL gives confident identifications (30-40% coverage) with 50 fmol of sample and the 4800 gives similar coverage with 10 fmol of sample. However high peptide loads are required to give a 117 reporter ion peak area of above four which appears to produce better quantification. In a real sample the individual proteins would be at unknown concentrations and span a large concentration range, so accurate quantification is called for at all concentrations.

### 3.4 Relative fragmentation efficiencies

**Fig. 4:**
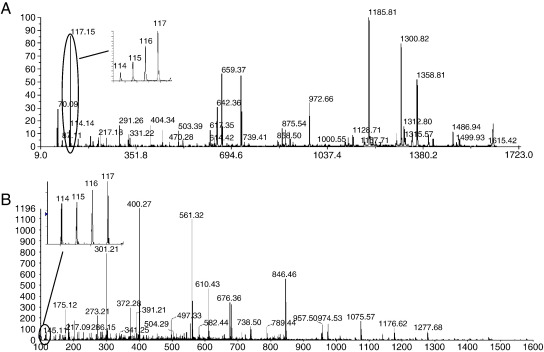
Spectra demonstrating the range of relative intensities seen for the iTRAQ reporter ions compared to *y* and *b* ion signal intensity. **A** nLC-MALDI MSMS spectrum for peptide ADDGRPFPQVIK (1630.9 *m*/*z*), **B** nLC-ESI MSMS spectrum for peptide GVVPLAGTNGETTTQGLDGLSER (806.4 *m*/*z*).

Examination of the MSMS spectra generated by the MALDI mass spectrometer showed that the reporter ions dominated the fragmentation spectra (Fig. 4A). In contrast, the nLC-ESI MSMS spectra, acquired at a collision energy (CE) set 10 V above the standard rolling CE used for peptide fragmentation, as recommended by the manufacturer, show b and y fragment ions as the most intense peaks, with the reporter ions of only moderate to low abundance (an example showing low abundance reporter ions is shown in Fig. 4B). Weise et al. [11] evaluated various peptide fragmentation spectra from nLC-ESI MSMS analysis on a QStar XL and noticed similar moderate to low abundances for the reporter ions. They showed that increasing the CE and changing the collision gas from nitrogen to argon improved the reporter ion abundance, and the accuracy of the peak area ratio, where the expected ratio was 2, from 1.6 (SD 15%) to 1.9 (SD 13%). However, increasing the CE resulted in lower intensity b and y ion fragments, particularly those above 800 Da, thus compromising the protein identification. The QStar Elite instrument, used by Kuzyk et al. [7], employs signal enhancement of the low mass reporter ion region in iTRAQ experiments, which may improve the accuracy of the reporter ion ratios in nLC-ESI MSMS data.

### 3.5 Precursor ion selection efficiency

Poor precursor ion selection efficiency could lead to the selection of a mixed precursor and hence mixed MSMS, which is likely to depress the ratio observed for those peptides showing up- (or down-) regulation, as most species in an experiment will show no change in expression. This was seen by Ow et al. [17] who showed reliable quantitation for a simple four protein mix but saw significant suppression of up-regulated reporter ion ratios when a complex mixture of proteins was present, which they attributed to mixed MSMS occurring due to mixed precursor ion selection.

Hu et al. [22] recommend an efficient LC routine to reduce complexity and eliminate co-eluting peptides, which they found to be a problem with the Applied Biosystems 4700 MALDI TOF/TOF Analyser, which has a relatively broad precursor ion selection window compared to the Applied Biosystems 4800 instrument. Indeed, in our experiment, the 4800 MALDI TOF/TOF Analyser gave more accurate ratios than the QStar XL instrument.

Certainly, within the nLC-ESI MSMS data set there were individual measurements, within the series of measurements for a particular *m*/*z*, which showed mixed MSMS where a more intense ion of similar *m*/*z* had ‘bled through’ into the MSMS of the low intensity ion of interest. With this particular peptide mix, visually apparent mixed MSMS occurred at a rate of less than one affected measurement per series of measurements for a particular *m*/*z* and always in measurements from the chromatographic tail. These peaks were removed by applying either the 117 peak area threshold or the chromatographic elution filter. However, it is interesting to note that the ‘iTRAQ analysis settings’ button in Analyst QS2.0 on the QStar Elite, used by Kuzyk et al. [7], isolates only the first (carbon12) isotope, rather than the whole isotope cluster as was selected in this work.

### 3.6 Analysis of nLC-MALDI MSMS data using Mascot

The reporter ion peak areas were calculated using Matrix Science's TS2Mascot software. The user flexibility in the Mascot quantification method configuration was investigated. Table 3 shows the advantage of using the weighted geometric mean (which gives more weight to the more highly represented peptides, and is recommended if the accuracy is limited by counting statistics) over the geometric mean and gives almost the predicted ratios with the combined MALDI data from the triplicate analyses, for both Ald and CAH. Table 3 also shows the ratios obtained when selecting peptides above the ‘homology threshold’ or the ‘identity threshold’. For these MALDI data there appears to be no benefit in selecting the higher Identity Ion Score threshold, which removes only three peptides.

### 3.7 Analysis of nLC-ESI MSMS data using Mascot

**Fig. 5:**
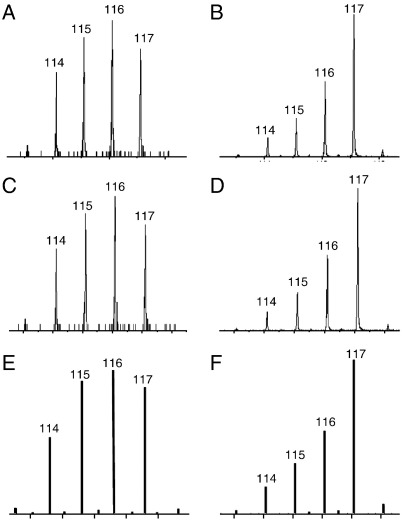
Comparison of raw data for nLC-ESI MSMS iTRAQ reporter ions with ProteinPilot Paragon and Mascot representations. **A** nLC-ESI MSMS cycle 1063, run 1, acquisition software (Analyst) and **B** nLC-ESI MSMS cycle 1535, run 1, acquisition software (Analyst). **C** cycle 1063 and **D** cycle 1535, ProteinPilot Paragon representation. **E** cycle 1063 and **F** cycle 1535, Mascot representation.

The fidelity of the reporter ion peak area extraction by Mascot can be seen by comparing Fig. 5 panels A and B to panels E and F (see also Supplementary Table 2).

Table 3 shows the weighted and normal geometric mean reporter ion ratios for Ald and CAH. The CAH ratios show very little change when the identity threshold was selected. This contrasted with the Ald results, where there was a significant improvement in the accuracy of the reporter ion ratios if the ‘at least identity’ threshold was used. Approximately 110 data points were removed, from the combined triplicate data set, by using the identity threshold instead of the homology threshold, and the data for four peptides assignments, from those found using the homology threshold, were excluded completely (Supplementary Table 3). Manual inspection of the MSMS for these four assignments did not suggest that they were false positives. The geometric mean for a further five peptide assignments improved upon removal of those matches below the identity threshold, whereas for two assignments there was a decrease in the geometric mean. Some data points are obvious outliers but there is no option to manually deselect such outliers, from the Mascot weighted average calculations and whilst Mascot uses statistical outlier removal methods, there was no change in the ratios obtained with this data set if the appropriate outlier removal method (Rosner's) was selected or no outlier removal method was selected.

**Fig. 6:**
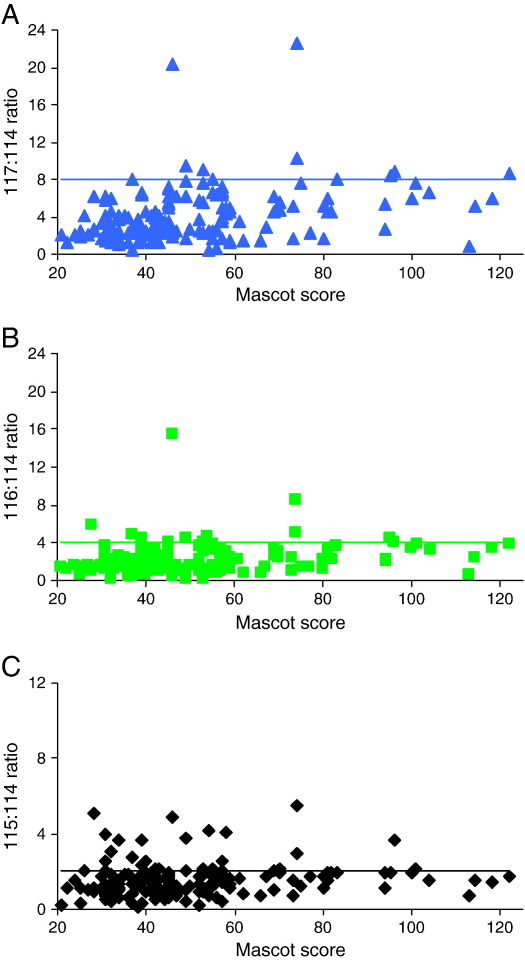
There is poor correlation between Mascot Ion Score and an accurate nLC-ESI MSMS reporter ion peak area ratio. A Reporter ion peak area ratio 117:114 from the triplicate analyses, calculated using peak areas from Analyst software, for each Ald peptide with an above ‘homology threshold’ Mascot Ion Score, a TIC above 12 for the most intense peak in the spectrum and a complete set of measureable reporter ions, plotted against Mascot Ion Score. The ratio is expected to be 8, as shown by horizontal line. **B** Reporter ion peak area ratio 116:114 from the triplicate analyses, calculated using peak areas from Analyst software, for each Ald peptide with an above ‘homology threshold’ Mascot Ion Score, a TIC above 12 for the most intense peak in the spectrum and a complete set of measureable reporter ions, plotted against Mascot Ion Score. The ratio is expected to be 4, as shown by horizontal line. **C** Reporter ion peak area ratio 115:114 from the triplicate analyses, calculated using peak areas from Analyst software, for each Ald peptide with an above ‘homology threshold’ Mascot Ion Score, a TIC above 12 for the most intense peak in the spectrum and a complete set of measureable reporter ions, plotted against Mascot Ion Score. The ratio is expected to be 2, as shown by horizontal line.

However, Fig. 6 shows that there is less correlation between the Mascot Ion Score for an individual peptide and the accuracy of the peak area ratios than was seen between the 117 peak area and the peak area ratios (Fig. 3D–F). Therefore, whilst the Mascot Ion Score and Expect values are an indication of the confidence with which the peaks seen in the MSMS match the peptide sequence, a high Ion Score does not necessarily imply that the quantification will be accurate.

As assessment of the Ald raw data (Fig. 3D–F) suggested that reliable quantification was more closely linked to the intensity of the reporter ions, the mechanisms available in Mascot to exclude peaks below a defined intensity were investigated. Mascot has no option to specifically remove reporter ion peaks below a particular threshold, but there is an option to globally remove MSMS signals below a certain intensity threshold either as a percentage of the most intense peak or an absolute intensity. The peak lists from the merged triplicate data sets were created for a range of peak intensity thresholds, to investigate whether removing the weaker signals from the peak lists (both reporter ions and y and b ions) improved the accuracy of quantification without having an adverse effect on the protein identification score. Table 4 shows the reporter ion ratios and Mascot scores for Ald and CAH obtained with a range of absolute intensity cut-offs for the merged triplicate data sets. Whilst it appears to be beneficial to remove some of the weakest data points, particularly for the Ald data, (compare no intensity threshold with an intensity threshold of 75) no specific intensity cut-off gives optimal ratios across all three reporter ion ratios for both proteins. However, intensity cut-off values in the range of 30 to 75 give the best overall improvement, with a value of 40 appearing to be the best global setting.

Supplementary Table 5 shows the equivalent reporter ion ratios and Mascot scores for Ald and CAH obtained with a range of percentage intensity cut-offs. Again it does appear to be useful to filter out the weakest peaks as seen with an intensity cut-off of 0.5%.

### 3.8 Analysis of nLC-ESI MSMS and nLC-MALDI MSMS data using ProteinPilot Paragon

One of the aims of the ProteinPilot Paragon method is to address the issue of which proteins, from the various isoforms in the database, can be inferred to be present from the peptides identified. To achieve this, it uses the Paragon [20] algorithm to determine the raw peptide identifications and then the ProGroup algorithm to group related redundant proteins with the identified protein. For the identification, although the algorithm ranks proteins based only on their unique peptides, it includes all the peptides in the coverage and in calculating the score. In contrast, for quantification only those peptides which are unique to a particular isoform are used in the calculation, as each isoform may be regulated independently and, hence, show different reporter ion ratios.

Fig. 1 indicates that ProteinPilot underestimates the degree of up-regulation of Ald and down-regulation of CAH to a greater extent than Mascot in both the nLC-ESI MSMS and nLC-MALDI MSMS analyses. The geometric means of the ratios, over the triplicate study, are shown in Table 5. For Ald, analysed by nLC-ESI MSMS, the ratios show large discrepancies. These are somewhat lower when analysed by nLC-MALDI MSMS. For the down-regulated protein, CAH, the degree of down-regulation was particularly underestimated by nLC-ESI MSMS analysis but within acceptable tolerance, with nLC-MALDI MSMS analysis. Fig. 5 C and D compared to A and B illustrates ProteinPilot Paragon's faithful peak extraction. The ratios obtained for the peptides that fulfilled the criteria of being selected by Mascot using the ‘at least homology’ threshold, having a total ion count for the most intense peak in the spectrum of 12 or above and a complete set of measureable reporter ion peak areas and were also selected by ProteinPilot Paragon are shown in Supplementary Tables 1 and 2. ProteinPilot Paragon uses the following criteria for selecting peptides for quantification. The peptide ID confidence must be > 1, the sum of the signal to noise ratio for all the peak pairs must be > 9 and the peptide must not be shared by more than one protein.

The elimination of peptides that are shared can result, in certain cases, in very few peptides being selected for the quantification, particularly for more common proteins, or if performing an “all species” search. In our sample this was particularly striking with Ald, where the rabbit and bovine sequences are very similar, resulting in 11 of the 13 peptides found matching both sequences and hence the quantification being performed on only the two unique rabbit peptides. This effect can be reduced by restricting the search to a specific species, however common proteins can have multiple entries in a database even within one species and so the quantification can be based on low peptide numbers.

The eliminated peptides can be manually added back into the quantification, by ticking a check box for each peptide. Adding in peptides with confidence > 95, which were previously eliminated because they were shared, in the case of Ald, increased the ratios from 1.91 to 2.10 (c.f. expected ratio of 2), 2.69 to 3.22 (c.f. expected ratio of 4) and 3.90 to 6.22 (c.f. expected ratio of 8) for the nLC-MALDI MSMS data set 1. This altered the 115:114 ratio from 5% underestimation to 5% overestimation, and reduced the underestimation for the 116:114 and 117:114 ratios from 33% and 51% respectively to 20% and 22%.

The power of the ProGroup algorithm is that it does not arbitrarily combine data from peptides that genuinely represent a closely related protein which shows a different pattern of up- or down-regulation to the related protein. However, it would be more powerful if it were capable of assessing whether peptide ratios are outliers and, hence, are likely to represent a differently regulated isoform, or whether they display a similar ratio to the peptides designated to the protein of interest and are therefore likely to have come from that protein.

### 3.9 Alternative routes to quantifying the iTRAQ reporter ion ratios

As there appears to be a tendency for the lower intensity reporter ions to give less accurate ratios, an averaging method that gave less weight to the lower intensity signals and more weight to the more intense signals might give more accurate ratios. This can be achieved by either summing the reporter ion peak areas from all the cycles and calculating the reporter ion ratios from the summed value or by summing the reporter ion peak areas measured for each peptide and calculating a ratio for each peptide from the summed peak areas, rather than averaging the individual ratios, and then calculating the geometric mean of those peptide ratios. A similar method is used by the Peaks algorithm [19] which initially calculates a weighting for each peptide ratio allowing peptide ratios from high quality spectra to be considered more reliable than those from spectra where the reporter ions are of low intensity. A related approach is used in the ‘ProRata’ algorithm from Pan et al. [23] designed for quantification, at the MS level, of SILAC [24] and ICAT [25] labelled peptides, where the peptide abundance ratio [26] is estimated from the ion intensities across the chromatographic peak and given a score based on a signal-to-noise ratio measure.

The simple summing methods described above, for the combined triplicate nLC-ESI MSMS data set for Ald gave ratios of 1.47 (115:114), 2.23 (116:114) and 3.83 (117:114) when all values were summed together and 1.50 (115:114), 2.17 (116:114) and 4.00 (117:114) when the peptides were summed individually. These ratios are closer to the expected values than those calculated by taking the geometric mean of each individual ratio (1.35 (115:114), 1.83 (116:114) and 3.29 (117:114)) but still show large discrepancies from the expected values, especially at higher ratios.

## 4 Conclusions

Although the results show good accuracy for the 4plex iTRAQ analysis at ratios of 1:1, there was a significant underestimation of the extent of protein up-regulation or down-regulation, particularly at higher ratios. This was very apparent in the nLC-ESI MSMS analysis, where the measured ratios for eight times up-regulation showed a 50% or greater underestimation depending on the software used for the analysis. If the degree of up- (or down-) regulation can be underestimated to this extent, large numbers of proteins where the degree of up- (or down-) regulation is measured as being below the significance threshold may actually be experiencing significant up- (or down-) regulation. For example, a protein that is actually up-regulated by a factor of 2.4 would only give a measurement of 1.2. Furthermore, the large standard deviations in ratio measurements also mitigate against successfully recording significant changes in expression levels when they are close to the threshold.

The reporter ion ratios measured by nLC-MALDI MSMS were much more accurate than those measured by nLC-ESI MSMS. One reason for this is likely to be the improved reporter ion ionization efficiency, relative to b and y ion fragmentation, seen in MALDI MSMS in our hands, compared to ESI MSMS. Another reason appears to be because in MALDI the reporter ions are measured, normally, only once for each peptide when it is at its chromatographic peak. Conversely in ESI analysis multiple acquisitions are made and those of weaker intensity, from the chromatographic tail, contribute less accurate ratios. We have shown that filtering out these peaks improves the accuracy of the ratios measured and demonstrated how some data filtering can be achieved within Mascot Daemon or the mascot.dll script for Sciex Analyst files. Unlike Ow et al. [17], we do not find widespread visually apparent co transmission of peptide precursors, resulting in mixed fragmentation and reporter ion ratios.

iTRAQ remains a useful quantitative method but the degree of up- or down-regulation measured should be treated with caution and proteins showing only small changes in expression level should be followed up using other methods as they may still be experiencing noteworthy changes in expression level.

## Figures and Tables

**Table 1:** Protein identifications by ProteinPilot Paragon and Mascot analysis of nLC-ESI MSMS and nLC-MALDI MSMS experiments for aldolase (Ald), carbonic anhydrase (CAH), bovine serum albumin (BSA) β-galactosidase (βGal), transferrin (TF), alcohol dehydrogenase (ADH), cytochrome C (cytoC) and lysozyme C (lysoC).

		**ESI**			**MALDI**			**ESI**			**MALDI**		
PP1	PP2	PP3	PP1	PP2	PP3	Mas1	Mas2	Mas3	Mas1	Mas2	Mas3
**Ald**	Score	31	46	37	28	22	13	985	1497	1153	987	792	645
P00883	% coverage	77	90	73	55	51	36	47	58	50	42	32	22
	unique pep	28	23	25	2^a^	2^a^	9	20	29	21	15	12	6
**CAH**	Score	37	35	36	36	30	25	573	940	619	1410	1132	856
P00915	% coverage	75	68	84	77	67	65	37	63	49	69	53	49
	unique pep	21	19	19	19	5^a^	13	12	17	11	18	16	10
**BSA**	Score	56	77	65	64	61	41	1343	2498	1863	845	1016	825
P02769	% coverage	83	82	74	62	71	46	48	76	57	20	23	18
	unique pep	38	7^a^	8^a^	6^a^	4^a^	4^a^	26	51	34	14	14	10
**βGal**	Score	50	72	62	55	63	49	940	1756	1527	1706	2017	1686
P00722	% coverage	56	67	61	47	48	42	20	45	39	25	30	27
	unique pep	5	43	43	28	38	30	20	36	32	23	28	23
**TF**	Score	62	92	79	73	66	50	1550	2889	2399	1478	1382	1216
Q29433	% coverage	80	80	73	64	59	52	47	67	61	26	21	21
	unique pep	46	51	48	43	35	28	38	62	45	20	19	15
**ADH**	Score	25	33	26	31	31	13	718	1295	832	1327	1081	456
P00330	% coverage	70	79	71	57	66	33	42	62	48	53	51	17
	unique pep	19	22	18	4^a^	18	7^a^	17	24	17	15	15	5
**CytoC**	Score	8	18	12	16	17	7	327	667	517	560	711	273
Q3LUG8	% coverage	83	75	69	61	66	47	51	70	69	56	65	41
	unique pep	9	14	6	9	7	6	8	16	9	9	13	5
**LysoC**	Score	9	19	15	14	17	14	368	696	606	399	448	393
P00698	% coverage	65	80	82	61	71	65	40	75	75	35	35	35
	unique pep	7	8	12	7	10	8	7	11	10	5	4	5
False	% Above Identity	–	–	–	–	–	–	1.19	3.48	0.61	5.71	8.99	11.90
Discovery	% Above Homology	–	–	–	–	–	–	9.61	9.94	2.38	9.51	14.49	18.12
Rate	5% Protein Level - Local	129	32	38				–	–	–	–	–	–

PP = ProteinPilot Paragon, Mas = Mascot, 1, 2, 3 refer to each of the triplicate analyses, unique pep = number of unique peptides identified.

a indicates where the number of unique peptides is artificially low due to the ProGroup algorithm treatment of redundant protein hits.

**Table 2:** Manually calculated geometric mean values derived from raw data using the triplicate merged data sets.

Ald	115:114 (exp ratio 2)	116:114 (exp ratio 4)	117:114 (exp ratio 8)
**MALDI** Geo mean (Geo SD) threshold = ‘homology’ score	1.74 (SD 1.28)	2.88 (SD 1.41)	5.69 (SD 1.39)
**MALDI** Geo mean (Geo SD) threshold = 20,000	1.98 (SD 1.18)	3.50 (SD 1.18)	7.39 (SD 1.16)
**ESI** Geo mean (Geo SD) threshold = ‘homology’ score	1.35 (SD 1.84)	1.83 (SD 1.93)	3.29 (SD 1.95)
**ESI** Geo mean (Geo SD) threshold = 4	1.66 (SD 1.23)	2.94 (SD 1.42)	5.22 (SD 1.62)
**ESI** Geo mean (Geo SD) threshold = above 50% chromatographic peak height	1.63 (SD 1.46)	2.67 (SD 1.76)	4.01 (SD 2.00)
**ESI** Geo mean (Geo SD) threshold = above 50% chromatographic peak height and 4	1.64 (SD 1.25)	2.98 (SD 1.50)	5.16 (SD 1.73)

Note: the geometric standard deviation is a factor such that the 95% confidence interval for the 115:114 ratio is from 1.36 to 2.23.

**Table 3:** Comparison of ratios from Mascot search for the ESI and MALDI-acquired merged triplicate data set calculated with weighted geometric and geometric means.

	115:114	116:114	117:114	115:114	116:114	117:114
Ald			CAH		
> Homology threshold MALDI						
weighted geometric mean	2.19 NN	3.91 NN	7.33 1.17	0.50 1.07	0.24 NN	0.11 1.09
geometric mean	1.74 NN	2.95 NN	5.71 1.35	0.49 1.27	0.25 NN	0.12 1.33

> Identity threshold MALDI						
weighted geometric mean	2.20 NN	3.92 NN	7.35 1.17	0.49 1.07	0.24 NN	0.11 NN
geometric mean	1.72 NN	2.93 NN	5.80 1.34	0.48 1.26	0.25 NN	0.12 NN

> Homology threshold ESI						
weighted geometric mean	1.46 NN	2.23 NN	3.80 NN	0.60 NN	0.35 NN	0.19 NN
geometric mean	1.32 NN	1.86 NN	3.23 NN	0.68 NN	0.46 NN	0.41 NN

> Identity threshold ESI						
weighted geometric mean	1.59 NN	2.68 NN	4.84 NN	0.59 NN	0.35 NN	0.20 NN
geometric mean	1.55 NN	2.10 NN	3.68 NN	0.64 NN	0.44 NN	0.36 NN

NN = not normal and indicates the ratios are not consistent with a normal distribution.

**Table 4:** Effect of applying Mascot absolute intensity cut-off thresholds to merged triplicate nLC-ESI-MSMS data set.

A Aldolase						
Abs int cut-off	Mascot threshold	No. pep used for quant (115:114, 116:114, 117:114)	115:114 (exp 2)	116:114 (exp 4)	117:114 (exp 8)	Mascot score, no. peptides (unique), coverage
0	Homol	179, 179, 185–21 uni	1.46	2.23	3.80	1761, 528 (32) pep, 68% cov
Ident	69, 71, 71–17 uni	1.59	2.68	4.84
10	Homol	57, 58, 57–18 uni	1.52	2.36	4.11	1531, 204 (32) pep, 68% cov
Ident	37, 38, 37–15 uni	1.62	2.73	4.95
20	Homol	51, 52, 50–19 uni	1.54	2.45	4.38	1469, 138 (29) pep, 56% cov
Ident	39, 39, 39–17 uni	1.66	2.87	5.23
30	Homol	37, 37, 37–12 uni	1.52	2.44	4.26	1409, 119 (28) pep, 55% cov
Ident	26, 26, 26–11 uni	1.61	2.86	5.25
40	Homol	27, 28, 28–9 uni	1.52	2.44	4.27	1374, 108 (27) pep, 55% cov
Ident	16, 16, 16–6 uni	1.68	3.06	5.68
50	Homol	18, 18, 18–8 uni	1.48	2.40	4.18	1359, 97 (27) pep, 55% cov
Ident	12, 12, 12–6 uni	1.65	3.07	5.63
75	Homol	12, 11, 12–5 uni	1.78	3.32	5.61	1214, 82 (23) pep, 54% cov
Ident	9, 9, 9–4 uni	1.83	3.38	6.00
100	Homol	9, 9, 9–4 uni	1.53	2.42	3.96	1176, 67 (23) pep, 54% cov
Ident	6, 6, 6–3 uni	1.78	3.38	5.78
150	Homol	3, 3, 3, −3 uni	1.40	2.11	3.18	902, 44 (19) pep, 47% cov
Ident	2, 2, 2, −2 uni	1.87	3.81	6.14


B Carbonic anhydrase						
Abs int Cut-off	Mascot threshold	No. pep used for quant (115:114, 116:114, 117:114)	115:114 (exp 0.5)	116:114 (exp 0.25)	117:114 (exp 0.125)	Mascot score, no. peptides (unique), coverage
0	Homol	270, 269, 262–15 uni	0.60	0.35	0.19	969, 535 (17) pep, 63% cov
Ident	183, 181, 180–12 uni	0.59	0.35	0.20
10	Homol	80, 77, 75–14 uni	0.59	0.34	0.18	880, 213 (17) pep, 63% cov
Ident	55, 50, 47–8 uni	0.59	0.33	0.17
20	Homol	66, 59, 44–10 uni	0.59	0.34	0.17	742, 123 (16) pep, 63% cov
Ident	48, 43, 30–9 uni	0.60	0.34	0.17
30	Homol	53, 43, 28–10 uni	0.60	0.34	0.17	673, 95 (13) pep, 49% cov
Ident	42, 34, 20–8 uni	0.60	0.34	0.17
40	Homol	39, 32, 18–10 uni	0.59	0.33	0.17	674, 79 (13) pep, 49% cov
Ident	32, 26, 14–9 uni	0.60	0.33	0.16
50	Homol	31, 22, 10–9 uni	0.60	0.33	0.17	647, 70 (12) pep, 49% cov
Ident	25, 18, 7–9 uni	0.60	0.33	0.16
75	Homol	27, 18, 12–8 uni	0.60	0.33	0.18	631, 64 (13) pep, 59% cov
Ident	19, 13, 8–6 uni	0.60	0.34	0.19
100	Homol	20, 12, 9–7 uni	0.60	0.34	0.17	512, 40 (10) pep, 42% cov
Ident	13, 8, 6–6 uni	0.60	0.34	0.17
150	Homol	11, 10, 5–6 uni	0.61	0.34	0.17	387, 30 (6) pep, 29% cov
Ident	8, 8, 4–3 uni	0.62	0.35	0.18

exp = expected value. Homol = Homology threshold. Ident = Identity threshold. uni = unique peptides.

**Table 5:** Protein Pilot Paragon algorithm ratios for nLC-MALDI-MSMS and nLC-ESI-MSMS merged triplicate data sets.

Ald	115:114 (exp ratio 2)	116:114 (exp ratio 4)	117:114 (exp ratio 8)
**nLC-ESI-MSMS**	1.43 (SD1.03)	1.91 (SD 1.12)	3.05 (SD 1.15)
**nLC-MALDI-MSMS**	1.83 (SD 1.04)	2.81 (SD 1.06)	4.83 (SD 1.20)
CAH	115:114 (exp ratio 0.5)	116:114 (exp ratio 0.25)	117:114 (exp ratio 0.125)

**nLC-ESI-MSMS**	0.62 (SD 1.01)	0.39 (SD 1.04)	0.25 (SD1.25)
**nLC-MALDI-MSMS**	0.49 (SD 1.04)	0.27 (SD 1.01)	0.14 (SD1.05)

## Appendix A Supplementary data

Table 1 Supplementary material Reporter ion peak areas and peak area ratios for the triplicate nLC-MALDI MSMS data sets, calculated from the raw data using Data Explorer and corrected for isotope impurities, with ratios calculated by Mascot and ProteinPilot Paragon for comparison.

Table 2 Supplementary material Reporter ion peak areas and peak area ratios for the triplicate nLC-ESI MSMS data sets, calculated from the raw data using Analyst and corrected for isotope impurities, with ratios calculated by Mascot and ProteinPilot Paragon for comparison.

Table 3 Supplementary material Mascot generated reporter ion ratios for nLC-ESI MSMS merged triplicate data sets.

Table 4 Supplementary material Ratios from Mascot for merged triplicate ESI datasets.

Supplementary Table 5 Effect of applying Mascot% intensity cut-off thresholds to merged triplicate nLC-ESI MSMS data set.
