# Supplementary material for: A comparison of the accuracy of iTRAQ quantification by nLC-ESI MSMS and nLC-MALDI MSMS methods
Source: J Proteomics. 2010 May 7;73(7):1391–403. doi: 10.1016/j.jprot.2010.03.003 (PMC2880794; doi:10.1016/j.jprot.2010.03.003)
Supplement: Table 1 Supplementary material — Reporter ion peak areas and peak area ratios for the triplicate nLC-MALDI MSMS data sets, calculated from the raw data using Data Explorer and corrected for isotope impurities, with ratios calculated by Mascot and ProteinPilot Paragon for comparison. [file mmc1.doc]

**Table 1 Supplementary Material: Reporter ion peak areas and peak area ratios for the triplicate nLC-MALDI MSMS data sets, calculated from the raw data using Data Explorer and corrected for isotope impurities, with ratios calculated by Mascot and ProteinPilot Paragon for comparison.**

| **Obs m/z** | **Run**  **no.** | **Mascot Ion Score** | **114**  **peak area** | **115**  **peak area** | **116**  **peak area** | **117**  **peak area** | **Calc.**  **115: 114** | **Calc.116: 114** | **Calc.117: 114** | **Mascot 115:114** | **Mascot 116:114** | **Mascot 117:114** | **PPilot**  **115:114** | **PPilot**  **116:114** | **PPilot**  **117:114** |
| --- | --- | --- | --- | --- | --- | --- | --- | --- | --- | --- | --- | --- | --- | --- | --- |
| 1188.71 | 1 | 83 | 14188 | 26345 | 43224 | 97058 | 1.86 | 3.05 | 6.84 | 1.90 | 3.25 | 6.93 | 1.91 | 3.25 | 6.98 |
| 1225.68 | 1 | 46 | 237 | 346 | 644 | 1537 | 1.46 | 2.72 | 6.51 | 1.73 | 3.37 | 7.22 | 1.74 | 3.37 | 7.26 |
| 1620.91 | 1 | 107 | 9236 | 17837 | 26233 | 59126 | 1.93 | 2.84 | 6.40 | 1.93 | 2.83 | 6.55 | 1.94 | 2.83 | 6.60 |
| 1630.97 | 1 | 89 | 9324 | 25860 | 43029 | 74280 | 2.77 | 4.62 | 7.97 | 2.56 | 4.53 | 7.76 | 2.58 | 4.54 | 7.81 |
| 1634.85 | 1 | 115 | 13914 | 27117 | 44483 | 86653 | 1.95 | 3.20 | 6.23 | 1.81 | 3.15 | 5.70 | 1.82 | 3.15 | 5.74 |
| 1836.06 | 1 | 126 | 22467 | 47896 | 83039 | 204322 | 2.13 | 3.70 | 9.09 | 2.12 | 3.81 | 9.11 | 2.13 | 3.81 | 9.18 |
| 1851.95 | 1 | 54 | 3851 | 7499 | 10633 | 19787 | 1.95 | 2.76 | 5.14 | 1.88 | 2.84 | 5.61 | 1.90 | 2.84 | 5.64 |
| 2267.26 | 1 | 60 | 1678 | 2786 | 3040 | 6063 | 1.66 | 1.81 | 3.61 | 1.83 | 1.60 | 3.92 | 1.85 | 1.60 | 3.95 |
| 2267.38 | 1 | 38 | 1101 | 1624 | 2662 | 3006 | 1.48 | 2.42 | 2.73 | 1.86 | 2.93 | 3.32 | 1.87 | 2.94 | 3.33 |
| 2416.36 | 1 | 224 | 5562 | 13315 | 24610 | 54195 | 2.39 | 4.42 | 9.74 | 2.33 | 4.11 | 8.34 | 2.35 | 4.11 | 8.40 |
| 1089.70 | 2 | 57 | 13640 | 25910 | 50126 | 99804 | 1.90 | 3.67 | 7.32 | 1.87 | 3.59 | 7.23 | 1.89 | 3.59 | 7.28 |
| 1188.69 | 2 | 67 | 585 | 556 | 602 | 1555 | 0.95 | 1.03 | 2.66 | 0.87 | 1.06 | 2.58 | 0.88 | 1.06 | 2.60 |
| 1403.87 | 2 | 67 | 812 | 1325 | 2504 | 4358 | 1.63 | 3.087 | 5.37 | 1.90 | 3.56 | 6.51 | 1.91 | 3.56 | 6.54 |
| 1630.91 | 2 | 93 | 5114 | 8859 | 16384 | 34730 | 1.73 | 3.20 | 6.79 | 1.44 | 2.61 | 4.43 | 1.45 | 2.61 | 4.45 |
| 1835.93 | 2 | 129 | 4233 | 7442 | 12688 | 30930 | 1.76 | 3.00 | 7.31 | 1.76 | 3.10 | 6.49 | 1.77 | 3.10 | 6.53 |
| 1992.01 | 2 | 97 | 1427 | 3231 | 4325 | 9153 | 2.26 | 3.03 | 6.42 | 1.98 | 2.75 | 6.73 | 2.00 | 2.75 | 6.78 |
| 2251.16 | 2 | 142 | 1646 | 3470 | 5919 | 9137 | 2.11 | 3.60 | 5.55 | 1.86 | 3.12 | 5.72 | 1.87 | 3.12 | 5.76 |
| 2251.20 | 2 | 106 | 1020 | 1080 | 2029 | 4737 | 1.06 | 1.99 | 4.64 | 0.95 | 2.41 | 4.69 | 0.95 | 2.41 | 4.71 |
| 2386.09 | 2 | 49 | 227 | 526 | 669 | 1263 | 2.32 | 2.94 | 5.56 | 2.15 | 3.01 | 5.51 | 2.16 | 3.02 | 5.54 |
| 1620.88 | 3 | 133 | 1138 | 1825 | 3158 | 6237 | 1.60 | 2.78 | 5.48 | 1.54 | 2.77 | 5.17 | 1.55 | 2.77 | 5.21 |
| 1630.89 | 3 | 89 | 10359 | 16504 | 38665 | 72308 | 1.59 | 3.73 | 6.98 | 1.74 | 3.81 | 6.65 | 1.75 | 3.81 | 6.69 |
| 1835.93 | 3 | 125 | 2911 | 4308 | 10332 | 16478 | 1.48 | 3.55 | 5.66 | 1.73 | 3.99 | 6.52 | 1.74 | 4.00 | 6.55 |
| 2251.20 | 3 | 169 | 2482 | 3381 | 9826 | 13467 | 1.36 | 3.96 | 5.43 | 1.41 | 3.56 | 5.58 | 1.42 | 3.57 | 5.61 |
| 2386.13 | 3 | 129 | 234 | 378 | 359 | 863 | 1.62 | 1.54 | 3.69 | 1.75 | 1.85 | 3.58 | 1.77 | 1.85 | 3.61 |
