# Supplementary material for: A comparison of the accuracy of iTRAQ quantification by nLC-ESI MSMS and nLC-MALDI MSMS methods
Source: J Proteomics. 2010 May 7;73(7):1391–403. doi: 10.1016/j.jprot.2010.03.003 (PMC2880794; doi:10.1016/j.jprot.2010.03.003)
Supplement: Table 2 Supplementary material — Reporter ion peak areas and peak area ratios for the triplicate nLC-ESI MSMS data sets, calculated from the raw data using Analyst and corrected for isotope impurities, with ratios calculated by Mascot and ProteinPilot Paragon for comparison. [file mmc2.doc]

**Table 2 Supplementary Material: Reporter ion peak areas and peak area ratios for the triplicate nLC-ESI MSMS data sets, calculated from the raw data using Analyst and corrected for isotope impurities, with ratios calculated by Mascot and ProteinPilot Paragon for comparison.**

| **Obs**  **m/z** | **Ch. state** | **Run**  **No.** | **Mascot Ion Score** | **114**  **peak area** | **115**  **peak area** | **116**  **peak area** | **117**  **peak area** | **Calc.115:**  **114** | **Calc.116:**  **114** | **Calc.117:**  **114** | **Mascot 115:114** | **Mascot 116:114** | **Mascot 117:114** | **PPilot**  **115:114** | **PPilot**  **116:114** | **PPilot**  **117:114** |
| --- | --- | --- | --- | --- | --- | --- | --- | --- | --- | --- | --- | --- | --- | --- | --- | --- |
| 436.80 | +2 | 1 | 45 | 1.56 | 2.98 | 4.96 | 8.72 | 1.91 | 3.19 | 5.60 | 1.84 | 2.92 | 5.45 | 1.81 | 2.89 | 5.22 |
| 436.80 | +2 | 1 | 45 | 1.45 | 2.67 | 4.23 | 7.84 | 1.85 | 2.92 | 5.42 | 1.90 | 3.18 | 5.56 | 1.77 | 2.93 | 5.16 |
| 436.80 | +2 | 1 | 44 | 1.01 | 1.64 | 2.46 | 2.79 | 1.63 | 2.45 | 2.77 | 1.62 | 2.44 | 2.75 | 1.70 | 2.45 | 2.74 |
| 594.84 | +2 | 1 | 57 | 2.66 | 4.97 | 9.09 | 17.41 | 1.87 | 3.41 | 6.53 | 1.85 | 3.41 | 6.49 | 1.94 | 3.62 | 6.74 |
| 594.84 | +2 | 1 | 57 | 5.40 | 10.82 | 18.90 | 33.90 | 2.00 | 3.50 | 6.27 | 1.95 | 3.43 | 6.12 | 1.96 | 3.38 | 6.16 |
| 594.84 | +2 | 1 | 57 | 2.40 | 4.37 | 8.48 | 16.16 | 1.82 | 3.53 | 6.73 | 1.81 | 3.53 | 6.69 | 1.90 | 3.46 | 6.54 |
| 594.84 | +2 | 1 | 53 | 0.91 | 1.84 | 2.86 | 5.02 | 2.02 | 3.14 | 5.51 | 2.00 | 3.13 | 5.48 | 1.85 | 2.87 | 5.07 |
| 594.84 | +2 | 1 | 57 | 0.47 | 0.80 | 0.97 | 1.85 | 1.71 | 2.06 | 3.94 | 1.70 | 2.06 | 3.91 | 1.67 | 2.65 | 3.83 |
| 594.84 | +2 | 1 | 57 | 0.66 | 0.93 | 1.48 | 2.58 | 1.42 | 2.52 | 3.92 | 1.41 | 2.25 | 3.90 | 1.44 | 2.34 | 3.94 |
| 613.36 | +2 | 1 | 55 | 0.50 | 1.05 | 1.94 | 3.97 | 2.09 | 3.88 | 7.93 | 2.07 | 4.14 | 7.87 | 2.13 | 4.19 | 7.82 |
| 613.36 | +2 | 1 | 53 | 0.62 | 1.34 | 1.95 | 5.6 | 2.15 | 3.12 | 8.99 | 2.08 | 4.66 | 8.85 | 2.00 | 4.10 | 7.72 |
| 452.94 | +2 | 1 | 42 | 0.85 | 1.51 | 1.51 | 1.97 | 1.77 | 1.78 | 2.32 | 1.76 | 1.78 | 2.31 | 1.71 | 1.82 | 2.24 |
| 810.96 | +2 | 1 | 101 | 1.17 | 2.45 | 4.62 | 8.97 | 2.09 | 3.94 | 7.64 | 2.08 | 3.94 | 7.59 | 2.10 | 3.91 | 7.70 |
| 810.96 | +2 | 1 | 70 | 1.09 | 2.31 | 3.36 | 5.13 | 2.11 | 3.07 | 4.69 | 2.10 | 3.07 | 4.66 | 1.89 | 2.73 | 4.10 |
| 540.99 | +3 | 1 | 81 | 0.88 | 1.68 | 2.63 | 3.92 | 1.91 | 3.00 | 4.47 | 1.90 | 2.99 | 4.44 | 1.76 | 2.78 | 4.13 |
| 543.96 | +3 | 1 | 45 | 0.17 | 0.11 | 0.24 | 0.56 | 0.61 | 1.39 | 3.24 | 0.61 | 1.39 | 3.22 | 1.12 | 1.73 | 2.84 |
| 543.96 | +3 | 1 | 39 | 0.09 | 0.21 | 0.39 | 0.56 | 2.38 | 4.44 | 6.40 | 2.37 | 4.43 | 6.35 | 1.16 | 2.08 | 2.83 |
| 544.32 | +3 | 1 | 35 | 4.36 | 8.12 | 10.66 | 17.60 | 1.86 | 2.45 | 4.04 | 1.85 | 2.44 | 4.00 | 1.88 | 2.44 | 4.04 |
| 817.91 | +3 | 1 | 83 | 1.87 | 3.71 | 7.01 | 15.09 | 1.98 | 3.75 | 8.07 | 1.97 | 3.75 | 8.01 | 2.03 | 3.77 | 8.36 |
| 817.91 | +3 | 1 | 73 | 1.56 | 2.30 | 3.66 | 7.90 | 1.48 | 2.35 | 5.06 | 1.47 | 2.34 | 5.02 | 1.66 | 2.35 | 5.02 |
| 817.91 | +3 | 1 | 55 | 0.94 | 1.59 | 1.48 | 2.32 | 1.69 | 1.58 | 2.47 | 1.67 | 1.57 | 2.45 | 1.68 | 1.63 | 2.41 |
| 612.67 | +3 | 1 | 61 | 1.17 | 1.91 | 2.70 | 4.13 | 1.64 | 2.31 | 3.55 | 1.63 | 2.31 | 3.53 | 1.53 | 2.11 | 3.25 |
| 612.67 | +3 | 1 | 62 | 1.53 | 1.25 | 1.37 | 2.17 | 0.82 | 0.90 | 1.42 | 0.82 | 0.91 | 1.41 | 0.84 | 1.07 | 1.43 |
| 618.00 | +3 | 1 | 49 | 1.64 | 1.72 | 1.99 | 2.75 | 1.05 | 1.21 | 1.67 | 1.04 | 1.21 | 1.66 | 1.04 | 1.20 | 1.65 |
| 618.00 | +3 | 1 | 43 | 16.44 | 18.35 | 17.15 | 21.91 | 1.12 | 1.04 | 1.33 | 1.11 | 1.04 | 1.32 | 1.13 | 1.07 | 1.30 |
| 756.43 | +3 | 1 | 82 | 0.62 | 1.18 | 1.40 | 2.74 | 1.92 | 2.27 | 4.46 | 1.91 | 2.26 | 4.58 | 1.29 | 2.06 | 3.10 |
| 806.11 | +3 | 1 | 113 | 2.80 | 2.13 | 1.66 | 2.53 | 0.76 | 0.59 | 0.90 | 0.76 | 0.59 | 0.90 | 0.76 | 0.71 | 0.87 |
| 436.79 | +2 | 2 | 45 | 0.77 | 1.30 | 2.7 | 5.40 | 1.70 | 3.53 | 7.05 | 1.69 | 3.53 | 7.00 | 1.69 | 3.50 | 6.97 |
| 436.79 | +2 | 2 | 47 | 0.08 | 0.06 | 0.13 | 0.20 | 0.78 | 1.65 | 2.44 | 0.78 | 1.64 | 2.43 |  | 2.09 | 2.38 |
| 436.79 | +2 | 2 | 42 | 0.03 | 0.05 | 0.11 | 0.16 | 1.47 | 3.31 | 4.51 | 1.46 | 3.30 | 4.48 |  |  |  |
| 436.79 | +2 | 2 | 42 | 0.06 | 0.06 | 0.10 | 0.21 | 1.09 | 1.84 | 3.80 | 1.09 | 1.84 | 3.77 |  |  |  |
| 436.79 | +2 | 2 | 43 | 0.08 | 0.06 | 0.11 | 0.19 | 0.72 | 1.33 | 2.35 | 0.72 | 1.33 | 2.38 |  |  | 2.82 |
| 545.36 | +2 | 2 | 53 | 1.21 | 2.20 | 4.86 | 9.23 | 1.82 | 4.02 | 7.64 | 1.81 | 4.02 | 7.59 | 1.79 | 3.99 | 7.56 |
| 545.36 | +2 | 2 | 52 | 0.08 | 0.02 | 0.12 | 0.21 | 0.20 | 1.47 | 2.55 | 0.21 | 1.46 | 2.54 |  | 1.51 | 2.48 |
| 545.36 | +2 | 2 | 52 | 0.06 | 0.10 | 0.12 | 0.34 | 1.70 | 2.10 | 5.80 | 1.68 | 2.10 | 5.76 |  |  | 5.55 |
| 613.34 | +2 | 2 | 57 | 0.57 | 0.84 | 1.91 | 4.01 | 1.47 | 3.36 | 7.09 | 1.47 | 3.37 | 7.04 | 1.51 | 3.42 | 7.24 |
| 452.94 | +3 | 2 | 45 | 0.34 | 0.34 | 0.47 | 1.17 | 1.00 | 1.39 | 3.44 | 0.99 | 1.39 | 3.42 | 0.98 | 1.36 | 3.30 |
| 810.97 | +2 | 2 | 122 | 0.23 | 0.40 | 0.89 | 1.94 | 1.76 | 3.92 | 8.52 | 1.75 | 3.92 | 8.47 | 1.99 | 3.84 | 8.46 |
| 810.97 | +2 | 2 | 66 | 0.67 | 0.47 | 0.59 | 0.99 | 0.70 | 0.88 | 1.49 | 0.70 | 0.88 | 1.48 | 0.71 | 0.91 | 1.47 |
| 810.97 | +2 | 2 | 100 | 0.09 | 0.17 | 0.30 | 0.53 | 1.90 | 3.44 | 6.02 | 1.91 | 3.43 | 5.99 | 1.73 | 2.80 | 5.13 |
| 810.97 | +2 | 2 | 94 | 0.06 | 0.13 | 0.15 | 0.34 | 1.96 | 2.33 | 5.36 | 1.95 | 2.33 | 5.32 |  |  | 5.73 |
| 810.97 | +2 | 2 | 55 | 0.11 | 0.04 | 0.04 | 0.19 | 1.21 | 1.07 | 1.67 | 1.26 | 1.12 | 1.74 | 1.20 | 1.20 | 1.74 |
| 810.97 | +2 | 2 | 52 | 0.01 | 0.05 | 0.03 | 0.08 | 4.20 | 4.70 | 0.32 | 1.27 | 3.55 | 2.54 |  |  |  |
| 810.97 | +2 | 2 | 54 | 0.04 | 0.03 | 0.00 | 0.00 | 0.96 | 0.14 | 2.23 | 4.21 | 3.58 | 0.37 |  |  |  |
| 810.97 | +2 | 2 | 53 | 0.06 | 0.04 | 0.05 | 0.03 | 0.65 | 0.87 | 0.52 | 0.96 | 0.15 | 2.21 |  |  |  |
| 810.97 | +2 | 2 | 56 | 0.02 | 0.03 | 0.09 | 0.06 | 1.27 | 3.57 | 2.55 | 0.65 | 0.86 | 0.52 |  |  |  |
| 543.97 | +3 | 2 | 74 | 0.06 | 0.30 | 0.47 | 1.25 | 5.47 | 8.61 | 22.66 | 5.41 | 8.61 | 22.46 | 4.58 | 7.19 | 18.83 |
| 544.32 | +3 | 2 | 39 | 0.60 | 1.12 | 1.98 | 3.92 | 1.87 | 3.32 | 6.57 | 1.86 | 3.32 | 6.56 | 1.85 | 3.29 | 6.47 |
| 544.32 | +3 | 2 | 37 | 0.38 | 1.06 | 1.83 | 3.09 | 2.78 | 4.80 | 8.09 | 2.76 | 4.79 | 8.09 | 2.77 | 4.64 | 8.41 |
| 817.92 | +2 | 2 | 104 | 0.09 | 0.14 | 0.29 | 0.59 | 1.57 | 3.29 | 6.58 | 1.56 | 3.29 | 6.53 | 1.62 | 3.22 | 6.20 |
| 593.92 | +3 | 2 | 70 | 0.48 | 0.92 | 1.21 | 2.63 | 1.90 | 2.51 | 5.45 | 1.89 | 2.51 | 5.41 | 1.88 | 2.68 | 5.53 |
| 612.66 | +3 | 2 | 59 | 0.14 | 0.41 | 0.70 | 1.42 | 2.91 | 5.03 | 10.17 | 2.89 | 5.03 | 10.10 | 2.85 | 5.31 | 9.70 |
| 612.66 | +3 | 2 | 59 | 0.13 | 0.22 | 0.17 | 0.37 | 1.75 | 1.35 | 2.86 | 1.73 | 1.35 | 2.84 | 1.68 | 1.40 | 2.89 |
| 664.70 | +3 | 2 | 96 | 0.36 | 0.59 | 0.86 | 1.59 | 1.64 | 2.40 | 4.47 | 1.49 | 2.30 | 4.60 | 1.61 | 2.46 | 4.30 |
| 664.70 | +3 | 2 | 94 | 0.38 | 0.50 | 0.88 | 1.83 | 1.32 | 2.32 | 4.82 | 1.49 | 2.30 | 4.60 | 1.33 | 2.34 | 4.77 |
| 751.10 | +3 | 2 | 57 | 0.10 | 0.39 | 0.44 | 0.93 | 3.71 | 4.16 | 8.89 | 3.67 | 4.21 | 8.81 | 3.43 | 5.60 | 8.72 |
| 751.10 | +3 | 2 | 38 | 0.09 | 0.10 | 0.18 | 0.24 | 1.08 | 1.95 | 2.63 | 1.07 | 1.95 | 2.61 |  | 2.17 | 3.31 |
| 751.10 | +3 | 2 | 34 | 0.07 | 0.03 | 0.08 | 0.11 | 0.41 | 1.07 | 1.56 | 0.41 | 1.07 | 1.55 |  |  |  |
| 751.10 | +3 | 2 | 46 | 0.07 | 0.09 | 0.05 | 0.17 | 1.23 | 0.66 | 2.27 | 1.22 | 0.52 | 2.26 |  |  |  |
| 751.10 | +3 | 2 | 41 | 0.05 | 0.06 | 0.07 | 0.05 | 1.14 | 1.34 | 0.98 | 1.14 | 1.13 | 0.98 |  |  |  |
| 756.42 | +3 | 2 | 80 | 0.06 | 0.05 | 0.02 | 0.10 | 0.86 | 0.43 | 1.86 | 0.85 | 0.43 | 1.85 |  |  |  |
| 756.42 | +3 | 2 | 37 | 0.08 | 0.07 | 0.10 | 0.09 | 0.93 | 1.18 | 1.18 | 0.92 | 1.18 | 1.17 |  |  |  |
| 777.48 | +3 | 2 | 32 | 0.08 | 0.14 | 0.18 | 0.43 | 1.71 | 2.15 | 5.11 | 1.70 | 2.15 | 5.07 |  | 2.82 | 6.60 |
| 796.03 | +3 | 2 | 75 | 0.08 | 0.02 | 0.03 | 0.02 | 0.26 | 0.44 | 0.32 | 0.26 | 0.44 | 0.32 |  |  |  |
| 796.03 | +3 | 2 | 45 | 0.03 | 0.01 | 0.01 | 0.04 | 0.43 | 0.26 | 1.19 | 0.43 | 0.26 | 1.18 |  |  |  |
| 1111.3 | +3 | 2 | 47 | 0.02 | 0.02 | 0.02 | 0.11 | 1.27 | 1.33 | 7.59 | 1.25 | 1.34 | 7.54 |  |  |  |
| 436.80 | +2 | 3 | 45 | 2.09 | 3.46 | 8.64 | 14.80 | 1.65 | 4.14 | 7.09 | 1.65 | 4.13 | 7.05 | 1.65 | 4.10 | 7.01 |
| 436.80 | +2 | 3 | 45 | 1.26 | 1.96 | 3.94 | 7.79 | 1.56 | 3.13 | 6.18 | 1.55 | 3.12 | 6.14 | 1.55 | 3.10 | 6.10 |
| 436.80 | +2 | 3 | 45 | 1.26 | 1.86 | 3.28 | 6.18 | 1.48 | 2.61 | 4.92 | 1.47 | 2.61 | 4.89 | 1.48 | 2.59 | 4.88 |
| 542.78 | +2 | 3 | 55 | 2.70 | 5.14 | 8.90 | 17.56 | 1.91 | 3.30 | 6.51 | 1.89 | 3.30 | 6.48 | 1.89 | 3.51 | 6.43 |
| 594.82 | +2 | 3 | 57 | 4.19 | 6.56 | 11.76 | 20.23 | 1.57 | 2.81 | 4.83 | 1.56 | 2.81 | 4.80 | 1.55 | 2.78 | 4.76 |
| 594.82 | +2 | 3 | 52 | 1.71 | 2.33 | 3.84 | 6.37 | 1.36 | 2.25 | 3.73 | 1.36 | 2.24 | 3.71 | 1.33 | 2.34 | 3.65 |
| 452.92 | +3 | 3 | 41 | 2.73 | 3.12 | 4.50 | 6.99 | 1.14 | 1.65 | 2.56 | 1.14 | 1.65 | 2.55 | 1.10 | 1.70 | 2.48 |
| 810.94 | +2 | 3 | 118 | 1.52 | 2.21 | 5.18 | 9.06 | 1.46 | 3.42 | 5.97 | 1.45 | 3.42 | 5.94 | 1.44 | 3.36 | 5.87 |
| 810.94 | +2 | 3 | 114 | 0.46 | 0.68 | 1.12 | 2.36 | 1.50 | 2.46 | 5.16 | 1.49 | 2.46 | 5.13 | 1.49 | 2.44 | 5.05 |
| 543.96 | +3 | 3 | 69 | 1.16 | 1.21 | 4.03 | 5.23 | 1.04 | 3.48 | 4.52 | 1.04 | 3.48 | 4.50 | 1.04 | 3.50 | 4.47 |
| 815.94 | +2 | 3 | 58 | 0.90 | 1.27 | 2.16 | 3.79 | 1.42 | 2.41 | 4.23 | 1.41 | 2.41 | 4.20 | 1.40 | 2.41 | 4.18 |
| 817.89 | +2 | 3 | 95 | 0.94 | 1.78 | 4.22 | 7.90 | 1.89 | 4.49 | 8.40 | 1.88 | 4.49 | 8.35 | 1.88 | 4.45 | 8.32 |
| 817.89 | +2 | 3 | 81 | 0.39 | 0.62 | 1.28 | 2.29 | 1.57 | 3.26 | 5.86 | 1.56 | 3.26 | 5.82 |  |  |  |
| 895.95 | +2 | 3 | 49 | 0.65 | 1.30 | 2.94 | 5.10 | 2.01 | 4.53 | 7.86 | 2.00 | 4.53 | 7.81 |  |  |  |
| 895.95 | +2 | 3 | 59 | 2.17 | 2.41 | 2.57 | 3.06 | 1.11 | 1.18 | 1.41 | 1.11 | 1.18 | 1.40 |  |  |  |
| 612.63 | +3 | 3 | 73 | 1.13 | 0.84 | 1.25 | 1.75 | 0.75 | 1.11 | 1.56 | 0.74 | 1.11 | 1.55 | 0.74 | 1.16 | 1.55 |
| 751.04 | +3 | 3 | 77 | 0.36 | 0.61 | 0.54 | 0.85 | 1.68 | 1.51 | 2.35 | 1.67 | 1.50 | 2.34 | 1.65 | 1.73 | 2.28 |
| 751.04 | +3 | 3 | 59 | 0.21 | 0.34 | 0.31 | 0.25 | 1.66 | 1.47 | 1.21 | 1.65 | 1.47 | 1.20 | 1.90 | 1.56 | 1.26 |
| 617.96 | +3 | 3 | 22 | 9.70 | 10.40 | 11.33 | 12.18 | 1.07 | 1.17 | 1.26 | 1.07 | 1.16 | 1.25 | 1.07 | 1.16 | 1.24 |
| 756.38 | +3 | 3 | 80 | 0.44 | 0.48 | 0.49 | 0.74 | 1.10 | 1.14 | 1.69 | 1.09 | 1.13 | 1.68 | 1.09 | 1.26 | 1.64 |
| 796.03 | +3 | 3 | 36 | 0.21 | 0.38 | 0.31 | 0.47 | 1.81 | 1.50 | 2.24 | 1.80 | 1.50 | 2.23 | 1.67 | 1.37 | 2.23 |
| 796.03 | +3 | 3 | 39 | 0.17 | 0.26 | 0.24 | 0.46 | 1.55 | 1.45 | 2.71 | 1.54 | 1.45 | 2.69 | 1.55 | 1.37 | 2.42 |
| 796.03 | +3 | 3 | 52 | 0.15 | 0.15 | 0.16 | 0.38 | 0.97 | 1.08 | 2.51 | 0.96 | 1.07 | 2.50 | 1.06 | 0.87 | 2.19 |
| 796.03 | +3 | 3 | 25 | 0.28 | 0.30 | 0.31 | 0.54 | 1.08 | 1.09 | 1.92 | 1.08 | 1.09 | 1.91 | 1.17 | 1.23 | 1.89 |
| 796.03 | +3 | 3 | 49 | 0.01 | 0.01 | 0.01 | 0.06 | 0.43 | 0.22 | 6.14 | 0.43 | 0.23 | 6.13 |  |  |  |
| 796.03 | +3 | 3 | 34 | 0.25 | 0.38 | 0.44 | 0.94 | 1.50 | 1.74 | 3.73 | 1.48 | 1.74 | 3.70 | 1.36 | 1.61 | 3.06 |
| 796.03 | +3 | 3 | 69 | 0.28 | 0.59 | 0.78 | 1.73 | 2.07 | 2.73 | 6.07 | 1.86 | 2.73 | 6.00 | 1.94 | 2.61 | 5.89 |
| 796.03 | +3 | 3 | 41 | 0.14 | 0.19 | 0.19 | 0.29 | 1.38 | 1.39 | 2.11 | 1.37 | 1.40 | 1.87 | 1.39 | 1.34 | 1.79 |
| 796.03 | +3 | 3 | 42 | 0.24 | 0.27 | 0.42 | 0.64 | 1.13 | 1.80 | 2.71 | 1.13 | 1.80 | 2.69 | 1.28 | 1.78 | 2.68 |
| 796.03 | +3 | 3 | 54 | 0.24 | 0.28 | 0.28 | 0.58 | 1.17 | 1.17 | 2.40 | 1.16 | 1.17 | 2.38 | 1.27 | 1.21 | 2.48 |
| 796.03 | +3 | 3 | 31 | 0.20 | 0.15 | 0.22 | 0.30 | 0.74 | 1.13 | 1.54 | 0.74 | 1.12 | 1.53 | 0.72 | 1.09 | 1.50 |
| 796.03 | +3 | 3 | 30 | 0.13 | 0.15 | 0.21 | 0.19 | 1.17 | 1.65 | 1.55 | 1.12 | 1.64 | 1.54 | 1.04 | 1.51 | 1.53 |
| 796.03 | +3 | 3 | 31 | 0.17 | 0.23 | 0.23 | 0.31 | 1.36 | 1.34 | 1.85 | 1.35 | 1.34 | 1.83 | 1.32 | 1.39 | 1.79 |
| 796.03 | +3 | 3 | 56 | 0.29 | 0.47 | 0.44 | 0.76 | 1.62 | 1.49 | 2.61 | 1.61 | 1.49 | 2.59 | 1.53 | 1.35 | 2.50 |
| 796.03 | +3 | 3 | 34 | 0.21 | 0.21 | 0.30 | 0.50 | 1.02 | 1.45 | 2.44 | 1.02 | 1.45 | 2.43 | 1.25 | 1.43 | 2.37 |
| 796.03 | +3 | 3 | 33 | 0.18 | 0.19 | 0.23 | 0.35 | 1.10 | 1.30 | 2.02 | 1.10 | 1.29 | 2.01 | 1.09 | 1.52 | 1.99 |
| 796.03 | +3 | 3 | 27 | 0.09 | 0.09 | 0.15 | 0.18 | 0.97 | 1.69 | 2.00 | 0.97 | 1.69 | 1.99 | 1.82 | 1.37 | 2.63 |
| 796.03 | +3 | 3 | 38 | 0.12 | 0.16 | 0.15 | 0.33 | 1.32 | 1.23 | 2.78 | 1.31 | 1.23 | 2.76 | 1.52 | 1.45 | 3.15 |
| 796.04 | +3 | 3 | 47 | 0.11 | 0.08 | 0.10 | 0.27 | 0.73 | 0.92 | 2.52 | 0.73 | 0.92 | 2.50 |  | 0.96 | 2.61 |
| 796.04 | +3 | 3 | 28 | 0.11 | 0.12 | 0.12 | 0.28 | 1.13 | 1.10 | 2.63 | 1.12 | 1.10 | 2.61 | 1.13 | 1.09 | 2.65 |
| 796.04 | +3 | 3 | 30 | 0.13 | 0.22 | 0.18 | 0.41 | 1.75 | 1.37 | 3.19 | 1.73 | 1.38 | 3.16 | 0.96 | 0.77 | 1.79 |
| 796.04 | +3 | 3 | 46 | 0.23 | 0.29 | 0.33 | 0.53 | 1.26 | 1.43 | 2.30 | 1.26 | 1.43 | 2.28 | 1.20 | 1.33 | 2.17 |
| 796.04 | +3 | 3 | 49 | 0.01 | 0.05 | 0.06 | 0.13 | 3.80 | 4.49 | 9.34 | 3.75 | 4.48 | 9.23 |  |  |  |
| 796.04 | +3 | 3 | 44 | 0.09 | 0.08 | 0.10 | 0.19 | 0.99 | 1.12 | 2.21 | 0.99 | 1.12 | 2.19 | 1.62 |  | 2.22 |
| 796.04 | +3 | 3 | 39 | 0.09 | 0.14 | 0.22 | 0.23 | 1.60 | 2.41 | 2.63 | 1.59 | 2.40 | 2.62 | 1.84 | 2.15 | 2.62 |
| 796.04 | +3 | 3 | 41 | 0.06 | 0.09 | 0.17 | 0.12 | 1.59 | 2.79 | 2.06 | 1.59 | 2.40 | 2.77 |  | 2.37 | 3.09 |
| 796.04 | +3 | 3 | 40 | 0.05 | 0.10 | 0.17 | 0.12 | 1.93 | 3.37 | 2.49 | 1.92 | 3.35 | 2.48 |  | 3.32 |  |
| 796.04 | +3 | 3 | 31 | 0.05 | 0.09 | 0.06 | 0.21 | 1.89 | 1.29 | 4.18 | 1.87 | 1.29 | 4.14 |  |  |  |
| 796.04 | +3 | 3 | 42 | 0.11 | 0.24 | 0.22 | 0.37 | 2.17 | 1.94 | 3.29 | 2.15 | 1.94 | 3.26 | 2.16 | 1.87 | 3.43 |
| 796.04 | +3 | 3 | 56 | 0.04 | 0.07 | 0.03 | 0.25 | 1.74 | 0.80 | 6.44 | 2.34 | 1.10 | 8.60 |  |  | 11.65 |
| 796.04 | +3 | 3 | 34 | 0.06 | 0.21 | 0.15 | 0.23 | 3.66 | 2.60 | 4.08 | 3.62 | 2.6 | 4.04 | 2.04 | 1.70 | 2.60 |
| 796.04 | +3 | 3 | 50 | 0.10 | 0.13 | 0.17 | 0.27 | 1.21 | 1.60 | 2.61 | 1.21 | 1.59 | 2.59 | 1.57 | 1.57 | 2.69 |
| 796.04 | +3 | 3 | 32 | 0.06 | 0.11 | 0.09 | 0.13 | 1.81 | 1.55 | 2.13 | 1.80 | 1.55 | 2.11 |  |  | 2.97 |
| 796.04 | +3 | 3 | 54 | 0.04 | 0.03 | 0.03 | 0.10 | 0.68 | 0.75 | 2.42 | 0.68 | 0.75 | 2.40 |  |  |  |
| 796.04 | +3 | 3 | 31 | 0.05 | 0.04 | 0.07 | 0.08 | 0.71 | 1.43 | 1.58 | 0.71 | 1.43 | 1.58 |  |  |  |
| 796.04 | +3 | 3 | 57 | 0.05 | 0.14 | 0.12 | 0.19 | 2.58 | 2.22 | 3.51 | 2.55 | 2.22 | 3.47 |  |  | 3.46 |
| 796.04 | +3 | 3 | 31 | 0.15 | 0.16 | 0.22 | 0.34 | 1.13 | 1.55 | 2.34 | 1.13 | 1.54 | 2.32 | 0.86 | 1.43 | 1.83 |
| 796.04 | +3 | 3 | 31 | 0.02 | 0.09 | 0.09 | 0.15 | 3.98 | 3.67 | 6.23 | 3.94 | 3.67 | 6.17 |  |  |  |
| 796.04 | +3 | 3 | 31 | 0.09 | 0.19 | 0.19 | 0.33 | 2.03 | 2.01 | 3.48 | 2.02 | 2.01 | 3.46 | 1.71 | 1.80 | 3.09 |
| 796.04 | +3 | 3 | 25 | 0.02 | 0.01 | 0.01 | 0.04 | 0.26 | 0.56 | 2.37 | 0.26 | 0.56 | 2.35 |  |  |  |
| 796.04 | +3 | 3 | 26 | 0.08 | 0.17 | 0.13 | 0.34 | 2.06 | 1.50 | 4.12 | 2.04 | 1.51 | 4.08 | 1.98 | 1.50 | 3.87 |
| 796.04 | +3 | 3 | 32 | 0.01 | 0.03 | 0.02 | 0.06 | 3.09 | 2.26 | 5.88 | 3.07 | 2.27 | 5.83 |  |  |  |
| 796.04 | +3 | 3 | 37 | 0.06 | 0.08 | 0.09 | 0.17 | 1.36 | 1.53 | 2.90 | 1.17 | 1.53 | 2.87 |  |  | 4.32 |
| 796.04 | +3 | 3 | 31 | 0.04 | 0.03 | 0.05 | 0.14 | 0.79 | 1.34 | 3.57 | 0.79 | 1.34 | 3.54 |  |  |  |
| 796.04 | +3 | 3 | 24 | 0.08 | 0.13 | 0.13 | 0.18 | 1.48 | 1.54 | 2.15 | 1.47 | 1.53 | 2.13 |  | 1.47 | 2.29 |
| 796.04 | +3 | 3 | 39 | 0.14 | 0.04 | 0.16 | 0.22 | 0.26 | 1.12 | 1.52 | 0.26 | 1.11 | 1.51 | 0.63 | 1.10 | 1.51 |
| 796.04 | +3 | 3 | 32 | 0.04 | 0.05 | 0.08 | 0.18 | 1.29 | 2.07 | 4.48 | 1.29 | 2.07 | 4.45 |  |  | 3.52 |
| 796.04 | +3 | 3 | 34 | 0.19 | 0.26 | 0.30 | 0.30 | 1.36 | 1.61 | 1.58 | 1.35 | 1.60 | 1.57 | 1.32 | 1.56 | 1.38 |
| 796.04 | +3 | 3 | 42 | 0.05 | 0.03 | 0.08 | 0.15 | 0.56 | 1.76 | 3.35 | 0.56 | 1.75 | 3.33 |  |  |  |
| 796.04 | +3 | 3 | 46 | 0.01 | 0.02 | 0.07 | 0.10 | 4.92 | 15.43 | 20.38 | 4.94 | 15.52 | 20.46 |  |  |  |
| 796.04 | +3 | 3 | 47 | 0.02 | 0.02 | 0.02 | 0.12 | 1.20 | 0.96 | 6.26 | 1.19 | 0.97 | 6.19 |  |  |  |
| 796.04 | +3 | 3 | 58 | 0.03 | 0.11 | 0.09 | 0.14 | 4.04 | 3.04 | 4.83 | 3.99 | 3.03 | 4.78 |  |  |  |
| 796.04 | +3 | 3 | 41 | 0.02 | 0.03 | 0.02 | 0.08 | 1.46 | 1.04 | 4.15 | 1.45 | 1.04 | 4.11 |  |  |  |
| 796.04 | +3 | 3 | 32 | 0.02 | 0.02 | 0.01 | 0.06 | 0.95 | 0.59 | 3.11 | 0.94 | 0.59 | 3.07 |  |  |  |
| 796.04 | +3 | 3 | 31 | 0.02 | 0.05 | 0.03 | 0.11 | 2.54 | 1.73 | 5.84 | 2.52 | 1.74 | 5.78 |  |  |  |
| 796.04 | +3 | 3 | 43 | 0.03 | 0.07 | 0.09 | 0.14 | 2.11 | 2.72 | 4.13 | 2.09 | 2.72 | 4.10 |  |  |  |
| 796.04 | +3 | 3 | 31 | 0.08 | 0.04 | 0.17 | 0.15 | 0.54 | 2.21 | 1.95 | 0.55 | 2.20 | 1.95 |  | 2.26 | 1.65 |
| 796.04 | +3 | 3 | 28 | 0.04 | 0.04 | 0.06 | 0.10 | 0.92 | 1.39 | 2.40 | 0.79 | 1.39 | 2.38 |  |  |  |
| 796.05 | +3 | 3 | 28 | 0.02 | 0.09 | 0.11 | 0.11 | 5.06 | 5.89 | 6.20 | 5.01 | 5.29 | 6.16 |  |  |  |
| 796.05 | +3 | 3 | 34 | 0.06 | 0.04 | 0.09 | 0.15 | 0.65 | 1.48 | 2.51 | 0.65 | 1.48 | 2.50 |  |  |  |
| 796.05 | +3 | 3 | 36 | 0.02 | 0.04 | 0.02 | 0.10 | 1.79 | 0.98 | 3.97 | 1.56 | 1.00 | 3.92 |  |  |  |
| 796.05 | +3 | 3 | 38 | 0.01 | 0.02 | 0.02 | 0.06 | 1.62 | 1.48 | 3.79 | 1.61 | 1.48 | 3.76 |  |  |  |
| 796.05 | +3 | 3 | 44 | 0.03 | 0.01 | 0.03 | 0.09 | 0.50 | 1.25 | 3.47 | 0.50 | 1.25 | 3.42 |  |  |  |
| 796.05 | +3 | 3 | 40 | 0.04 | 0.10 | 0.11 | 0.18 | 2.51 | 2.72 | 4.57 | 2.49 | 2.72 | 4.53 |  |  | 4.67 |
| 796.05 | +3 | 3 | 39 | 0.02 | 0.03 | 0.05 | 0.05 | 1.42 | 2.42 | 2.52 | 1.42 | 2.41 | 2.50 |  |  |  |
| 796.05 | +3 | 3 | 37 | 0.07 | 0.06 | 0.16 | 0.15 | 0.82 | 2.21 | 2.19 | 0.82 | 2.20 | 2.18 | 1.87 | 2.60 | 2.78 |
| 796.05 | +3 | 3 | 39 | 0.01 | 0.04 | 0.02 | 0.06 | 3.70 | 1.68 | 6.52 | 3.66 | 1.70 | 6.46 |  |  |  |
| 796.05 | +3 | 3 | 21 | 0.05 | 0.01 | 0.06 | 0.09 | 0.23 | 1.37 | 1.98 | 0.23 | 1.36 | 1.97 |  |  |  |
| 796.05 | +3 | 3 | 40 | 0.02 | 0.01 | 0.01 | 0.03 | 0.94 | 0.54 | 2.05 | 0.94 | 0.54 | 2.03 |  |  |  |
| 796.06 | +3 | 3 | 36 | 0.04 | 0.02 | 0.02 | 0.05 | 0.56 | 0.42 | 1.27 | 0.56 | 0.42 | 1.26 |  |  |  |
| 796.06 | +3 | 3 | 42 | 0.02 | 0.03 | 0.06 | 0.05 | 1.65 | 3.19 | 2.47 | 1.65 | 3.18 | 2.46 |  |  |  |
| 796.06 | +3 | 3 | 31 | 0.01 | 0.02 | 0.02 | 0.02 | 2.53 | 2.43 | 2.03 | 2.51 | 2.42 | 2.02 |  |  |  |
| 796.06 | +3 | 3 | 38 | 0.02 | 0.01 | 0.04 | 0.02 | 0.12 | 1.98 | 1.18 | 0.13 | 1.96 | 1.18 |  |  |  |
