# Supplementary material for: A comparison of the accuracy of iTRAQ quantification by nLC-ESI MSMS and nLC-MALDI MSMS methods
Source: J Proteomics. 2010 May 7;73(7):1391–403. doi: 10.1016/j.jprot.2010.03.003 (PMC2880794; doi:10.1016/j.jprot.2010.03.003)
Supplement: Table 3 Supplementary material — Mascot generated reporter ion ratios for nLC-ESI MSMS merged triplicate data sets. [file mmc3.doc]

**Table 3 Supplementary Material: Mascot generated reporter ion ratios for nLC-ESI MSMS merged triplicate data sets**

| **m/z obs** | **Mr (calc)** | **115:114** | **116:114** | **117:114** | **Geo mean**  **& SD^h^**  **115:114** | **Geo mean**  **& SD^h^**  **116:114** | **Geo mean**  **& SD^h^**  **117:114** | **Geo mean**  **& SD^i^**  **115:114** | **Geo mean**  **& SD^i^**  **116:114** | **Geo mean**  **& SD^i^**  **117:114** |
| --- | --- | --- | --- | --- | --- | --- | --- | --- | --- | --- |
| 436.807 | 871.574 | 0.78 | 1.64 | 2.43 | 1.37 (1.39) | 2.59 (1.42) | 4.40 (1.50) | - | - | - |
| 436.786 |  | 1.47 | 2.61 | 4.89 |  |  |  |  |  |  |
| 436.787 |  | 1.55 | 3.12 | 6.14 |  |  |  |  |  |  |
| 436.791 |  | 1.65 | 4.13 | 7.05 |  |  |  |  |  |  |
| 436.803 |  | 1.46 | 3.30 | 4.48 |  |  |  |  |  |  |
| 436.803 |  | 1.69 | 3.53 | 7.00 |  |  |  |  |  |  |
| 436.808 |  | 0.72 | 1.33 | 2.38 |  |  |  |  |  |  |
| 436.810 |  | 1.09 | 1.84 | 3.77 |  |  |  |  |  |  |
| 436.799 |  | 1.90 | 3.18 | 5.56 |  |  |  |  |  |  |
| 436.804 |  | 1.84 | 2.92 | 5.45 |  |  |  |  |  |  |
| 436.8091 |  | 1.62 | 2.44 | 2.75 |  |  |  |  |  |  |
| *542.780 | 1083.579 | 1.89 | 3.30 | 6.48 | 1.89 | 3.30 | 6.48 | 1.89 | 3.30 | 6.48 |
| *545.354 | 1088.680 | 1.81 | 4.02 | 7.59 | 0.86 (3.43) | 2.31 (1.67) | 4.81 (1.77) | 1.81 | 4.02 | 7.59 |
| 545.359 |  | 0.21 | 1.46 | 2.54 |  |  |  |  |  |  |
| 545.361 |  | 1.68 | 2.10 | 5.76 |  |  |  |  |  |  |
| *594.815 | 1187.663 | 1.56 | 2.81 | 4.80 | 1.69 (1.6) | 2.80 (1.25) | 5.01 (1.28) | 1.69 (1.6) | 2.80 (1.25) | 5.01 (1.28) |
| *594.823 |  | 1.36 | 2.24 | 3.71 |  |  |  |  |  |  |
| *594.810 |  | 1.95 | 3.43 | 6.12 |  |  |  |  |  |  |
| *594.837 |  | 1.81 | 3.53 | 6.69 |  |  |  |  |  |  |
| *594.841 |  | 1.85 | 3.41 | 6.49 |  |  |  |  |  |  |
| *594.850 |  | 2.00 | 3.13 | 5.48 |  |  |  |  |  |  |
| *594.853 |  | 1.41 | 2.25 | 3.90 |  |  |  |  |  |  |
| *594.860 |  | 1.69 | 2.06 | 3.91 |  |  |  |  |  |  |
| *613.344 | 1224.660 | 1.47 | 3.37 | 7.04 | 1.85 (1.22) | 4.02 (1.18) | 7.89 (1.12) | 1.74 (1.27) | 3.73 (1.16) | 7.45 (1.08) |
| *613.3517 |  | 2.07 | 4.14 | 7.87 |  |  |  |  |  |  |
| 613.3566 |  | 2.08 | 4.66 | 8.85 |  |  |  |  |  |  |
| 452.940 | 1355.777 | 0.99 | 1.39 | 3.41 | 1.26 (1.35) | 1.60 (1.14) | 2.72 (1.23) | - | - | - |
| 452.919 |  | 1.14 | 1.65 | 2.55 |  |  |  |  |  |  |
| 452.941 |  | 1.76 | 1.78 | 2.31 |  |  |  |  |  |  |
| *810.967 | 1619.897 | 1.75 | 3.92 | 8.47 | 1.52 (1.62) | 1.99 (2.52) | 2.94 (2.64) | 1.52 (1.62) | 1.99 (2.52) | 2.94 (2.64) |
| *810.921 |  | 1.45 | 3.42 | 5.94 |  |  |  |  |  |  |
| *810.937 |  | 1.49 | 2.46 | 5.13 |  |  |  |  |  |  |
| *810.965 |  | 2.08 | 3.93 | 7.59 |  |  |  |  |  |  |
| *810.969 |  | 1.91 | 3.43 | 5.99 |  |  |  |  |  |  |
| *810.969 |  | 0.70 | 0.88 | 1.48 |  |  |  |  |  |  |
| *540.982 |  | 2.10 | 3.07 | 4.66 |  |  |  |  |  |  |
| *810.971 |  | 1.26 | 1.12 | 1.74 |  |  |  |  |  |  |
| *810.971 |  | 1.94 | 2.33 | 5.32 |  |  |  |  |  |  |
| *540.985 |  | 1.90 | 2.99 | 4.44 |  |  |  |  |  |  |
| *810.975 |  | 0.65 | 0.86 | 0.52 |  |  |  |  |  |  |
| *810.979 |  | 0.96 | 0.15 | 2.21 |  |  |  |  |  |  |
| *810.984 |  | 4.21 | 3.58 | 0.37 |  |  |  |  |  |  |
| *810.986 |  | 1.27 | 3.55 | 2.54 |  |  |  |  |  |  |
| *543.974 | 1628.888 | 5.41 | 8.61 | 22.46 | 1.69 (2.60) | 3.69 (2.13) | 6.74 (2.34) | 2.38 (3.20) | 5.48 (1.90) | 10.05 (3.12) |
| *543.955 |  | 1.04 | 3.48 | 4.50 |  |  |  |  |  |  |
| 543.986 |  | 0.61 | 1.39 | 3.22 |  |  |  |  |  |  |
| 543.987 |  | 2.37 | 4.43 | 6.35 |  |  |  |  |  |  |
| 815.944 | 1629.908 | 1.41 | 2.41 | 4.20 | 1.91 (1.32) | 3.11 (1.38) | 5.47 (1.41) | - | - | - |
| 544.317 |  | 1.86 | 3.32 | 6.56 |  |  |  |  |  |  |
| 544.318 |  | 1.85 | 2.44 | 4.00 |  |  |  |  |  |  |
| 544.320 |  | 2.76 | 4.79 | 8.09 |  |  |  |  |  |  |
| *817.921 | 1633.803 | 1.56 | 3.29 | 6.53 | 1.67 (1.12) | 2.95 (1.45) | 5.61 (1.57) | 1.67 (1.12) | 2.95 (1.45) | 5.61 (1.57) |
| *817.892 |  | 1.88 | 4.49 | 8.35 |  |  |  |  |  |  |
| *817.894 |  | 1.56 | 3.26 | 5.82 |  |  |  |  |  |  |
| *817.914 |  | 1.97 | 3.75 | 8.01 |  |  |  |  |  |  |
| *817.923 |  | 1.47 | 2.35 | 5.02 |  |  |  |  |  |  |
| *817.930 |  | 1.67 | 1.57 | 2.45 |  |  |  |  |  |  |
| *593.019 | 1775.999 | 1.89 | 2.51 | 5.41 | 1.89 | 2.51 | 5.41 | 1.89 | 2.51 | 5.41 |
| 895.945 | 1789.904 | 2.00 | 4.53 | 7.81 | 1.49 (1.52) | 2.31 (2.59) | 3.31 (3.37) | 1.11 | 1.18 | 1.40 |
| *895.951 |  | 1.11 | 1.18 | 1.40 |  |  |  |  |  |  |
| *612.664 | 1834.937 | 2.89 | 5.03 | 10.10 | 1.38 (1.76) | 1.73 (1.99) | 2.94 (2.21) | 1.38 (1.76) | 1.73 (1.99) | 2.94 (2.21) |
| *612.631 |  | 0.74 | 1.11 | 1.55 |  |  |  |  |  |  |
| *612.668 |  | 1.73 | 1.35 | 2.84 |  |  |  |  |  |  |
| *612.657 |  | 1.63 | 2.31 | 3.53 |  |  |  |  |  |  |
| *612.669 |  | 0.82 | 0.91 | 1.41 |  |  |  |  |  |  |
| 617.963 | 1850.932 | 1.07 | 1.16 | 1.25 | 1.07 (1.03) | 1.14 (1.08) | 1.40 (1.16) | - | - | - |
| 617.997 |  | 1.11 | 1.04 | 1.32 |  |  |  |  |  |  |
| 618.001 |  | 1.04 | 1.21 | 1.66 |  |  |  |  |  |  |
| *664.701 | 1991.038 | 1.49 | 2.30 | 4.60 | 1.49 | 2.30 | 4.60 | 1.49 | 2.30 | 4.60 |
| *751.046 | 2250.191 | 1.67 | 1.50 | 2.34 | 1.30 (1.93) | 1.42 (1.89) | 2.16 (2.05) | 1.35 (2.22) | 1.81 (1.68) | 2.51 (2.16) |
| *751.047 |  | 1.65 | 1.47 | 1.20 |  |  |  |  |  |  |
| 751.092 |  | 1.14 | 1.13 | 0.98 |  |  |  |  |  |  |
| *751.093 |  | 0.41 | 1.07 | 1.55 |  |  |  |  |  |  |
| *751.093 |  | 1.07 | 1.95 | 2.61 |  |  |  |  |  |  |
| 751.093 |  | 1.22 | 0.52 | 2.26 |  |  |  |  |  |  |
| *751.084 |  | 3.67 | 4.21 | 8.81 |  |  |  |  |  |  |
| *756.420 | 2266.186 | 1.91 | 2.26 | 4.58 | 1.13 (1.44) | 1.07 (1.98) | 2.02 (1.79) | 1.44 (1.48) | 1.60 (1.63) | 2.78 (2.03) |
| *756.378 |  | 1.09 | 1.13 | 1.68 |  |  |  |  |  |  |
| 756.419 |  | 0.85 | 0.43 | 1.85 |  |  |  |  |  |  |
| 756.425 |  | 0.92 | 1.178 | 1.17 |  |  |  |  |  |  |
| *777.478 | 2329.358 | 1.70 | 2.146 | 5.07 | 1.70 | 2.15 | 5.07 | 1.70 | 2.15 | 5.07 |
| *796.027 | 2385.136 | 1.86 | 2.734 | 6.00 | 1.23 (2.20) | 1.58 (1.99) | 2.89 (2.02) | 1.57 (1.84) | 1.73 (1.84) | 3.23 (2.09) |
| 796.027 |  | 1.48 | 1.736 | 3.70 |  |  |  |  |  |  |
| 796.029 |  | 1.37 | 1.396 | 1.87 |  |  |  |  |  |  |
| 796.029 |  | 1.13 | 1.797 | 2.69 |  |  |  |  |  |  |
| *796.030 |  | 1.16 | 1.165 | 2.38 |  |  |  |  |  |  |
| 796.030 |  | 0.74 | 1.124 | 1.53 |  |  |  |  |  |  |
| 796.031 |  | 1.12 | 1.642 | 1.54 |  |  |  |  |  |  |
| 796.031 |  | 1.80 | 1.495 | 2.23 |  |  |  |  |  |  |
| 796.031 |  | 1.08 | 1.089 | 1.91 |  |  |  |  |  |  |
| 796.031 |  | 1.35 | 1.337 | 1.83 |  |  |  |  |  |  |
| *796.031 |  | 1.61 | 1.493 | 2.59 |  |  |  |  |  |  |
| 796.033 |  | 1.02 | 1.445 | 2.43 |  |  |  |  |  |  |
| 796.034 |  | 1.88 | 5.151 | -0.24 |  |  |  |  |  |  |
| 796.034 |  | 1.10 | 1.294 | 2.01 |  |  |  |  |  |  |
| 796.034 |  | 0.97 | 1.687 | 1.99 |  |  |  |  |  |  |
| 796.034 |  | 1.31 | 1.232 | 2.76 |  |  |  |  |  |  |
| *796.035 |  | 0.73 | 0.924 | 2.50 |  |  |  |  |  |  |
| 796.035 |  | 1.12 | 1.102 | 2.61 |  |  |  |  |  |  |
| *796.035 |  | 0.96 | 1.072 | 2.50 |  |  |  |  |  |  |
| 796.036 |  | 1.73 | 1.375 | 3.16 |  |  |  |  |  |  |
| *796.036 |  | 1.26 | 1.427 | 2.28 |  |  |  |  |  |  |
| *796.036 |  | 3.75 | 4.477 | 9.23 |  |  |  |  |  |  |
| 796.037 |  | 0.99 | 1.120 | 2.19 |  |  |  |  |  |  |
| 796.037 |  | 1.59 | 2.402 | 2.62 |  |  |  |  |  |  |
| 796.038 |  | 1.59 | 2.397 | 2.77 |  |  |  |  |  |  |
| 796.039 |  | 1.92 | 3.349 | 2.48 |  |  |  |  |  |  |
| 796.039 |  | 1.87 | 1.293 | 4.14 |  |  |  |  |  |  |
| 796.039 |  | 2.15 | 1.938 | 3.26 |  |  |  |  |  |  |
| *796.040 |  | 2.34 | 1.098 | 8.60 |  |  |  |  |  |  |
| 796.040 |  | 3.62 | 2.600 | 4.04 |  |  |  |  |  |  |
| *796.040 |  | 1.21 | 1.592 | 2.59 |  |  |  |  |  |  |
| 796.040 |  | 1.80 | 1.550 | 2.11 |  |  |  |  |  |  |
| *796.040 |  | 0.68 | 0.753 | 2.40 |  |  |  |  |  |  |
| 796.040 |  | 0.71 | 1.426 | 1.58 |  |  |  |  |  |  |
| *796.040 |  | 2.55 | 2.215 | 3.47 |  |  |  |  |  |  |
| 796.041 |  | -0.09 | 0.856 | 3.77 |  |  |  |  |  |  |
| 796.041 |  | 1.13 | 1.544 | 2.32 |  |  |  |  |  |  |
| 796.041 |  | 3.94 | 3.665 | 6.17 |  |  |  |  |  |  |
| 796.041 |  | 2.02 | 2.006 | 3.46 |  |  |  |  |  |  |
| 796.041 |  | 0.26 | 0.560 | 2.35 |  |  |  |  |  |  |
| 796.041 |  | 2.04 | 1.505 | 4.08 |  |  |  |  |  |  |
| 796.041 |  | 1.54 | 1.450 | 2.69 |  |  |  |  |  |  |
| 796.041 |  | 3.07 | 2.269 | 5.83 |  |  |  |  |  |  |
| 796.041 |  | 0.97 | -0.069 | 0.24 |  |  |  |  |  |  |
| 796.042 |  | 1.17 | 1.534 | 2.87 |  |  |  |  |  |  |
| 796.042 |  | 0.79 | 1.342 | 3.54 |  |  |  |  |  |  |
| 796.042 |  | 1.47 | 1.534 | 2.13 |  |  |  |  |  |  |
| 796.042 |  | 0.26 | 1.113 | 1.51 |  |  |  |  |  |  |
| *796.042 |  | 1.90 | 4.006 | 0.85 |  |  |  |  |  |  |
| 796.042 |  | 1.29 | 2.071 | 4.45 |  |  |  |  |  |  |
| 796.042 |  | 1.35 | 1.599 | 1.57 |  |  |  |  |  |  |
| 796.042 |  | 0.56 | 1.754 | 3.33 |  |  |  |  |  |  |
| 796.043 |  | 4.94 | 15.522 | 20.46 |  |  |  |  |  |  |
| *796.043 |  | 1.19 | 0.971 | 6.19 |  |  |  |  |  |  |
| *796.043 |  | 3.99 | 3.025 | 4.78 |  |  |  |  |  |  |
| 796.043 |  | 1.45 | 1.040 | 4.11 |  |  |  |  |  |  |
| 796.044 |  | 0.94 | 0.590 | 3.07 |  |  |  |  |  |  |
| 796.044 |  | 2.52 | 1.742 | 5.78 |  |  |  |  |  |  |
| 796.044 |  | 2.09 | 2.718 | 4.10 |  |  |  |  |  |  |
| 796.044 |  | 0.55 | 2.196 | 1.95 |  |  |  |  |  |  |
| 796.044 |  | 0.79 | 1.289 | 2.38 |  |  |  |  |  |  |
| 796.044 |  | 2.86 | 2.629 | 5.59 |  |  |  |  |  |  |
| 796.044 |  | 4.25 | -0.725 | 12.02 |  |  |  |  |  |  |
| 796.045 |  | 8.94 | 14.194 | 18.43 |  |  |  |  |  |  |
| *796.045 |  | -0.12 | 1.861 | 3.76 |  |  |  |  |  |  |
| 796.045 |  | 0.18 | 0.451 | 1.00 |  |  |  |  |  |  |
| 796.045 |  | 5.01 | 5.285 | 6.16 |  |  |  |  |  |  |
| 796.046 |  | 0.65 | 1.476 | 2.50 |  |  |  |  |  |  |
| 796.046 |  | 0.93 | 1.035 | 2.02 |  |  |  |  |  |  |
| 796.046 |  | 1.56 | 0.996 | 3.92 |  |  |  |  |  |  |
| 796.046 |  | -0.07 | -0.284 | 7.04 |  |  |  |  |  |  |
| 796.046 |  | 1.61 | 1.484 | 3.76 |  |  |  |  |  |  |
| 796.046 |  | 0.34 | 0.991 | -0.05 |  |  |  |  |  |  |
| *796.047 |  | 0.67 | 2.735 | 2.14 |  |  |  |  |  |  |
| 796.047 |  | 0.50 | 1.253 | 3.42 |  |  |  |  |  |  |
| 796.047 |  | 2.49 | 2.717 | 4.53 |  |  |  |  |  |  |
| 796.048 |  | 0.93 | 0.578 | 9.80 |  |  |  |  |  |  |
| 796.048 |  | 1.42 | 2.411 | 2.50 |  |  |  |  |  |  |
| 796.048 |  | 0.96 | -0.183 | 3.10 |  |  |  |  |  |  |
| *796.048 |  | 4.15 | 4.806 | 10.80 |  |  |  |  |  |  |
| 796.048 |  | 0.82 | 2.201 | 2.18 |  |  |  |  |  |  |
| 796.049 |  | 3.66 | 1.698 | 6.46 |  |  |  |  |  |  |
| 796.051 |  | 0.23 | 1.364 | 1.97 |  |  |  |  |  |  |
| 796.052 |  | 0.96 | -0.225 | 4.13 |  |  |  |  |  |  |
| 796.053 |  | 0.94 | 0.543 | 2.03 |  |  |  |  |  |  |
| 796.054 |  | -0.06 | -0.022 | 0.51 |  |  |  |  |  |  |
| 796.055 |  | 0.56 | 0.421 | 1.26 |  |  |  |  |  |  |
| 796.055 |  | 8.04 | -0.643 | 4.67 |  |  |  |  |  |  |
| *796.056 |  | -0.08 | 0.725 | 0.73 |  |  |  |  |  |  |
| 796.057 |  | 1.65 | 3.183 | 2.46 |  |  |  |  |  |  |
| 796.057 |  | 0.90 | 1.872 | 3.00 |  |  |  |  |  |  |
| 796.058 |  | 2.51 | 2.424 | 2.02 |  |  |  |  |  |  |
| 796.059 |  | 1.26 | 1.493 | 3.74 |  |  |  |  |  |  |
| 796.059 |  | 0.53 | 2.899 | 2.94 |  |  |  |  |  |  |
| 796.059 |  | 0.45 | -0.277 | 6.16 |  |  |  |  |  |  |
| 796.094 |  | -0.09 | 0.887 | 3.00 |  |  |  |  |  |  |
| 796.060 |  | 1.49 | -0.214 | 3.14 |  |  |  |  |  |  |
| 796.061 |  | 0.13 | 1.964 | 1.18 |  |  |  |  |  |  |
| 796.062 |  | -0.60 | -0.042 | 1.01 |  |  |  |  |  |  |
| 796.069 |  | 0.43 | 0.261 | 1.18 |  |  |  |  |  |  |
| 796.070 |  | 0.45 | -0.195 | 4.10 |  |  |  |  |  |  |
| 796.070 |  | -0.18 | 3.877 | 2.84 |  |  |  |  |  |  |
| 796.071 |  | .26 | 0.439 | 0.32 |  |  |  |  |  |  |
| 796.072 |  | 0.43 | 0.234 | 6.13 |  |  |  |  |  |  |
| *806.102 | 2415.236 | 0.76 | 0.593 | 0.90 | 0.76 | 0.59 | 0.90 | 0.76 | 0.59 | 0.90 |
| *1111.275 | 3330.755 | 1.25 | 1.341 | 7.54 | 1.25 | 1.34 | 7.54 | 1.25 | 1.34 | 7.54 |

*denotes those measurements remaining upon selection of the Identity threshold

^h^ peptides above the Homology threshold selected

^i^ peptides above the Identity threshold selected
