# Supplementary material for: A comparison of the accuracy of iTRAQ quantification by nLC-ESI MSMS and nLC-MALDI MSMS methods
Source: J Proteomics. 2010 May 7;73(7):1391–403. doi: 10.1016/j.jprot.2010.03.003 (PMC2880794; doi:10.1016/j.jprot.2010.03.003)
Supplement: Table 4 Supplementary material — Ratios from Mascot for merged triplicate ESI datasets. [file mmc4.doc]

**Table 4 Supplementary Material: Ratios from Mascot for merged triplicate ESI datasets**

| m/z obs | Mr (calc) | 115:114 | 116:114 | 117:114 | Geo mean  & SD^h^  115:114 | Geo mean  & SD^h^  116:114 | Geo mean  & SD^h^  117:114 | Geo mean  & SD^i^  115:114 | Geo mean  & SD^i^  116:114 | Geo mean  & SD^i^  117:114 |
| --- | --- | --- | --- | --- | --- | --- | --- | --- | --- | --- |
| 436.807 | 871.574 | 0.779 | 1.644 | 2.431 | 1.371 (1.394) | 2.593 (1.421) | 4.403 (1.498) | - | - | - |
| 436.786 |  | 1.470 | 2.608 | 4.886 |  |  |  |  |  |  |
| 436.787 |  | 1.546 | 3.124 | 6.140 |  |  |  |  |  |  |
| 436.791 |  | 1.646 | 4.132 | 7.048 |  |  |  |  |  |  |
| 436.803 |  | 1.458 | 3.296 | 4.477 |  |  |  |  |  |  |
| 436.803 |  | 1.689 | 3.529 | 7.003 |  |  |  |  |  |  |
| 436.808 |  | 0.723 | 1.328 | 2.378 |  |  |  |  |  |  |
| 436.810 |  | 1.090 | 1.841 | 3.772 |  |  |  |  |  |  |
| 436.799 |  | 1.902 | 3.180 | 5.557 |  |  |  |  |  |  |
| 436.804 |  | 1.836 | 2.917 | 5.451 |  |  |  |  |  |  |
| 436.809 |  | 1.624 | 2.439 | 2.752 |  |  |  |  |  |  |
| *542.780 | 1083.579 | 1.892 | 3.297 | 6.476 | 1.892 | 3.297 | 6.476 | 1.892 | 3.297 | 6.476 |
| *545.354 | 1088.680 | 1.806 | 4.018 | 7.590 | 0.855 (3.433) | 2.312 (1.669) | 4.805 (1.767) | 1.806 | 4.018 | 7.590 |
| 545.359 |  | 0.206 | 1.462 | 2.540 |  |  |  |  |  |  |
| 545.361 |  | 1.682 | 2.103 | 5.755 |  |  |  |  |  |  |
| *594.815 | 1187.663 | 1.559 | 2.805 | 4.803 | 1.690 (1.158) | 2.798 (1.246) | 5.007 (1.276) | 1.690 (1.158) | 2.798 (1.246) | 5.007 (1.276) |
| *594.823 |  | 1.356 | 2.241 | 3.707 |  |  |  |  |  |  |
| *594.810 |  | 1.953 | 3.432 | 6.123 |  |  |  |  |  |  |
| *594.837 |  | 1.809 | 3.529 | 6.693 |  |  |  |  |  |  |
| *594.841 |  | 1.854 | 3.408 | 6.492 |  |  |  |  |  |  |
| *594.850 |  | 2.004 | 3.132 | 5.476 |  |  |  |  |  |  |
| *594.853 |  | 1.414 | 2.249 | 3.898 |  |  |  |  |  |  |
| *594.860 |  | 1.695 | 2.058 | 3.905 |  |  |  |  |  |  |
| *613.344 | 1224.660 | 1.467 | 3.365 | 7.044 | 1.849 (1.222) | 4.018 (1.179) | 7.888 (1.121) | 1.741 (1.274) | 3.732 (1.158) | 7.455 (1.081) |
| *613.352 |  | 2.067 | 4.139 | 7.869 |  |  |  |  |  |  |
| 613.357 |  | 2.083 | 4.657 | 8.854 |  |  |  |  |  |  |
| 452.940 | 1355.777 | 0.990 | 1.389 | 3.415 | 1.257 (1.350) | 1.599 (1.136) | 2.717 (1.227) | - | - | - |
| 452.919 |  | 1.139 | 1.652 | 2.547 |  |  |  |  |  |  |
| 452.941 |  | 1.761 | 1.781 | 2.305 |  |  |  |  |  |  |
| *810.967 | 1619.897 | 1.749 | 3.916 | 8.470 | 1.516 (1.620) | 1.987 (2.523) | 2.939 (2.635) | 1.516 (1.620) | 1.987 (2.523) | 2.939 (2.635) |
| *810.921 |  | 1.450 | 3.417 | 5.944 |  |  |  |  |  |  |
| *810.937 |  | 1.486 | 2.459 | 5.128 |  |  |  |  |  |  |
| *810.965 |  | 2.075 | 3.935 | 7.589 |  |  |  |  |  |  |
| *810.969 |  | 1.912 | 3.430 | 5.985 |  |  |  |  |  |  |
| *810.969 |  | 0.697 | 0.879 | 1.483 |  |  |  |  |  |  |
| *540.982 |  | 2.096 | 3.070 | 4.657 |  |  |  |  |  |  |
| *810.971 |  | 1.259 | 1.121 | 1.738 |  |  |  |  |  |  |
| *810.971 |  | 1.947 | 2.331 | 5.318 |  |  |  |  |  |  |
| *540.985 |  | 1.898 | 2.992 | 4.443 |  |  |  |  |  |  |
| *810.975 |  | 0.653 | 0.864 | 0.517 |  |  |  |  |  |  |
| *810.979 |  | 0.955 | 0.146 | 2.205 |  |  |  |  |  |  |
| *810.984 |  | 4.205 | 3.577 | 0.372 |  |  |  |  |  |  |
| *810.986 |  | 1.273 | 3.548 | 2.541 |  |  |  |  |  |  |
| *543.974 | 1628.888 | 5.410 | 8.614 | 22.455 | 1.690 (2.601) | 3.686 (2.126) | 6.744 (2.336) | 2.377 (3.201) | 5.477 (1.897) | 10.051 (3.117) |
| *543.955 |  | 1.044 | 3.483 | 4.499 |  |  |  |  |  |  |
| 543.986 |  | 0.609 | 1.391 | 3.224 |  |  |  |  |  |  |
| 543.987 |  | 2.369 | 4.425 | 6.352 |  |  |  |  |  |  |
| 815.944 | 1629.908 | 1.409 | 2.407 | 4.201 | 1.912 (1.319) | 3.107 (1.383) | 5.465 (1.409) | - | - | - |
| 544.317 |  | 1.860 | 3.319 | 6.558 |  |  |  |  |  |  |
| 544.318 |  | 1.847 | 2.436 | 4.003 |  |  |  |  |  |  |
| 544.320 |  | 2.762 | 4.788 | 8.089 |  |  |  |  |  |  |
| *817.921 | 1633.803 | 1.555 | 3.286 | 6.532 | 1.675 (1.123) | 2.953 (2.588) | 5.613 (1.568) | 1.675 (1.123) | 2.953 (2.588) | 5.613 (1.568) |
| *817.892 |  | 1.880 | 4.485 | 8.353 |  |  |  |  |  |  |
| *817.894 |  | 1.562 | 3.256 | 5.822 |  |  |  |  |  |  |
| *817.914 |  | 1.969 | 3.749 | 8.013 |  |  |  |  |  |  |
| *817.923 |  | 1.466 | 2.345 | 5.022 |  |  |  |  |  |  |
| *817.930 |  | 1.674 | 1.573 | 2.447 |  |  |  |  |  |  |
| *593.019 | 1775.999 | 1.885 | 2.512 | 5.411 | 1.885 | 2.512 | 5.411 | 1.885 | 2.512 | 5.411 |
| 895.945 | 1789.904 | 1.999 | 4.525 | 7.814 | 1.486 (1.521) | 2.310 (2.588) | 3.309 (3.371) |  |  |  |
| *895.951 | 1789.904 | 1.105 | 1.179 | 1.401 |  |  |  | 1.105 | 1.179 | 1.401 |
| *612.664 | 1834.937 | 2.886 | 5.026 | 10.100 | 1.377 (1.759) | 1.734 (1.992) | 2.943 (2.207) | 1.377 (1.759) | 1.734 (1.992) | 2.943 (2.207) |
| *612.631 |  | 0.744 | 1.110 | 1.547 |  |  |  |  |  |  |
| *612.668 |  | 1.731 | 1.347 | 2.836 |  |  |  |  |  |  |
| *612.657 |  | 1.628 | 2.308 | 3.528 |  |  |  |  |  |  |
| *612.669 |  | 0.819 | 0.905 | 1.413 |  |  |  |  |  |  |
| 617.963 | 1850.932 | 1.068 | 1.164 | 1.247 | 1.072 (1.032) | 1.135 (1.081) | 1.399 (1.165) | - | - | - |
| 617.997 |  | 1.109 | 1.040 | 1.321 |  |  |  |  |  |  |
| 618.001 |  | 1.041 | 1.209 | 1.663 |  |  |  |  |  |  |
| *664.701 | 1991.038 | 1.493 | 2.304 | 4.601 | 1.493 | 2.304 | 4.601 | 1.493 | 2.304 | 4.601 |
| *751.046 | 2250.191 | 1.666 | 1.504 | 2.336 | 1.297 (1.926) | 1.415 (1.886) | 2.163 (2.051) | 1.347 (2.222) | 1.808 (1.679) | 2.513 (2.155) |
| *751.047 |  | 1.647 | 1.465 | 1.199 |  |  |  |  |  |  |
| 751.092 |  | 1.137 | 1.127 | 0.981 |  |  |  |  |  |  |
| *751.093 |  | 0.411 | 1.070 | 1.554 |  |  |  |  |  |  |
| *751.093 |  | 1.073 | 1.946 | 2.612 |  |  |  |  |  |  |
| 751.093 |  | 1.223 | 0.523 | 2.256 |  |  |  |  |  |  |
| *751.084 |  | 3.669 | 4.210 | 8.809 |  |  |  |  |  |  |
| *756.420 | 2266.186 | 1.906 | 2.257 | 4.583 | 1.131 (1.438) | 1.067 (1.978) | 2.021 (1.787) | 1.444 (1.481) | 1.598 (1.629) | 2.776 (2.032) |
| *756.378 |  | 1.094 | 1.132 | 1.682 |  |  |  |  |  |  |
| 756.419 |  | 0.850 | 0.431 | 1.850 |  |  |  |  |  |  |
| 756.425 |  | 0.924 | 1.178 | 1.169 |  |  |  |  |  |  |
| *777.478 | 2329.358 | 1.697 | 2.146 | 5.074 | 1.697 | 2.146 | 5.074 | 1.697 | 2.146 | 5.074 |
| *796.027 | 2385.136 | 1.857 | 2.734 | 6.002 | 1.228 (2.203) | 1.578 (1.985) | 2.892 (2.024) | 1.572 (1.841) | 1.725 (1.837) | 3.227 (2.087) |
| 796.027 |  | 1.482 | 1.736 | 3.702 |  |  |  |  |  |  |
| 796.029 |  | 1.368 | 1.396 | 1.866 |  |  |  |  |  |  |
| 796.029 |  | 1.126 | 1.797 | 2.694 |  |  |  |  |  |  |
| *796.030 |  | 1.163 | 1.165 | 2.384 |  |  |  |  |  |  |
| 796.030 |  | 0.740 | 1.124 | 1.529 |  |  |  |  |  |  |
| 796.031 |  | 1.119 | 1.642 | 1.538 |  |  |  |  |  |  |
| 796.031 |  | 1.797 | 1.495 | 2.226 |  |  |  |  |  |  |
| 796.031 |  | 1.075 | 1.089 | 1.906 |  |  |  |  |  |  |
| 796.031 |  | 1.352 | 1.337 | 1.833 |  |  |  |  |  |  |
| *796.031 |  | 1.611 | 1.493 | 2.586 |  |  |  |  |  |  |
| 796.033 |  | 1.016 | 1.445 | 2.427 |  |  |  |  |  |  |
| 796.034 |  | 1.881 | 5.151 | -0.239 |  |  |  |  |  |  |
| 796.034 |  | 1.099 | 1.294 | 2.008 |  |  |  |  |  |  |
| 796.034 |  | 0.966 | 1.687 | 1.987 |  |  |  |  |  |  |
| 796.034 |  | 1.314 | 1.232 | 2.756 |  |  |  |  |  |  |
| *796.035 |  | 0.732 | 0.924 | 2.501 |  |  |  |  |  |  |
| 796.035 |  | 1.124 | 1.102 | 2.610 |  |  |  |  |  |  |
| *796.035 |  | 0.960 | 1.072 | 2.495 |  |  |  |  |  |  |
| 796.036 |  | 1.730 | 1.375 | 3.159 |  |  |  |  |  |  |
| *796.036 |  | 1.257 | 1.427 | 2.280 |  |  |  |  |  |  |
| *796.036 |  | 3.745 | 4.477 | 9.233 |  |  |  |  |  |  |
| 796.037 |  | 0.985 | 1.120 | 2.193 |  |  |  |  |  |  |
| 796.037 |  | 1.590 | 2.402 | 2.619 |  |  |  |  |  |  |
| 796.038 |  | 1.592 | 2.397 | 2.766 |  |  |  |  |  |  |
| 796.039 |  | 1.923 | 3.349 | 2.477 |  |  |  |  |  |  |
| 796.039 |  | 1.873 | 1.293 | 4.143 |  |  |  |  |  |  |
| 796.039 |  | 2.153 | 1.938 | 3.262 |  |  |  |  |  |  |
| *796.040 |  | 2.340 | 1.098 | 8.598 |  |  |  |  |  |  |
| 796.040 |  | 3.621 | 2.600 | 4.035 |  |  |  |  |  |  |
| *796.040 |  | 1.209 | 1.592 | 2.593 |  |  |  |  |  |  |
| 796.040 |  | 1.797 | 1.550 | 2.112 |  |  |  |  |  |  |
| *796.040 |  | 0.676 | 0.753 | 2.403 |  |  |  |  |  |  |
| 796.040 |  | 0.710 | 1.426 | 1.578 |  |  |  |  |  |  |
| *796.040 |  | 2.552 | 2.215 | 3.474 |  |  |  |  |  |  |
| 796.041 |  | -0.093 | 0.856 | 3.765 |  |  |  |  |  |  |
| 796.041 |  | 1.128 | 1.544 | 2.321 |  |  |  |  |  |  |
| 796.041 |  | 3.940 | 3.665 | 6.173 |  |  |  |  |  |  |
| 796.041 |  | 2.016 | 2.006 | 3.455 |  |  |  |  |  |  |
| 796.041 |  | 0.256 | 0.560 | 2.349 |  |  |  |  |  |  |
| 796.041 |  | 2.036 | 1.505 | 4.081 |  |  |  |  |  |  |
| 796.041 |  | 1.535 | 1.450 | 2.694 |  |  |  |  |  |  |
| 796.041 |  | 3.066 | 2.269 | 5.825 |  |  |  |  |  |  |
| 796.041 |  | 0.965 | -0.069 | 0.238 |  |  |  |  |  |  |
| 796.042 |  | 1.173 | 1.534 | 2.867 |  |  |  |  |  |  |
| 796.042 |  | 0.785 | 1.342 | 3.539 |  |  |  |  |  |  |
| 796.042 |  | 1.470 | 1.534 | 2.134 |  |  |  |  |  |  |
| 796.042 |  | 0.264 | 1.113 | 1.514 |  |  |  |  |  |  |
| *796.042 |  | 1.896 | 4.006 | 0.852 |  |  |  |  |  |  |
| 796.042 |  | 1.285 | 2.071 | 4.452 |  |  |  |  |  |  |
| 796.042 |  | 1.354 | 1.599 | 1.566 |  |  |  |  |  |  |
| 796.042 |  | 0.557 | 1.754 | 3.331 |  |  |  |  |  |  |
| 796.043 |  | 4.943 | 15.522 | 20.461 |  |  |  |  |  |  |
| *796.043 |  | 1.185 | 0.971 | 6.192 |  |  |  |  |  |  |
| *796.043 |  | 3.994 | 3.025 | 4.776 |  |  |  |  |  |  |
| 796.043 |  | 1.445 | 1.040 | 4.107 |  |  |  |  |  |  |
| 796.044 |  | 0.939 | 0.590 | 3.074 |  |  |  |  |  |  |
| 796.044 |  | 2.517 | 1.742 | 5.782 |  |  |  |  |  |  |
| 796.044 |  | 2.094 | 2.718 | 4.102 |  |  |  |  |  |  |
| 796.044 |  | 0.547 | 2.196 | 1.949 |  |  |  |  |  |  |
| 796.044 |  | 0.786 | 1.289 | 2.380 |  |  |  |  |  |  |
| 796.044 |  | 2.855 | 2.629 | 5.585 |  |  |  |  |  |  |
| 796.044 |  | 4.247 | -0.725 | 12.015 |  |  |  |  |  |  |
| 796.045 |  | 8.940 | 14.194 | 18.431 |  |  |  |  |  |  |
| *796.045 |  | -0.123 | 1.861 | 3.762 |  |  |  |  |  |  |
| 796.045 |  | 0.178 | 0.451 | 0.998 |  |  |  |  |  |  |
| 796.045 |  | 5.013 | 5.285 | 6.155 |  |  |  |  |  |  |
| 796.046 |  | 0.653 | 1.476 | 2.499 |  |  |  |  |  |  |
| 796.046 |  | 0.929 | 1.035 | 2.015 |  |  |  |  |  |  |
| 796.046 |  | 1.560 | 0.996 | 3.923 |  |  |  |  |  |  |
| 796.046 |  | -0.066 | -0.284 | 7.042 |  |  |  |  |  |  |
| 796.046 |  | 1.612 | 1.484 | 3.762 |  |  |  |  |  |  |
| 796.046 |  | 0.343 | 0.991 | -0.046 |  |  |  |  |  |  |
| *796.047 |  | 0.667 | 2.735 | 2.137 |  |  |  |  |  |  |
| 796.047 |  | 0.502 | 1.253 | 3.422 |  |  |  |  |  |  |
| 796.047 |  | 2.486 | 2.717 | 4.527 |  |  |  |  |  |  |
| 796.048 |  | 0.930 | 0.578 | 9.804 |  |  |  |  |  |  |
| 796.048 |  | 1.416 | 2.411 | 2.497 |  |  |  |  |  |  |
| 796.048 |  | 0.957 | -0.183 | 3.096 |  |  |  |  |  |  |
| *796.048 |  | 4.145 | 4.806 | 10.797 |  |  |  |  |  |  |
| 796.048 |  | 0.817 | 2.201 | 2.179 |  |  |  |  |  |  |
| 796.049 |  | 3.659 | 1.698 | 6.456 |  |  |  |  |  |  |
| 796.051 |  | 0.230 | 1.364 | 1.971 |  |  |  |  |  |  |
| 796.052 |  | 0.963 | -0.225 | 4.131 |  |  |  |  |  |  |
| 796.053 |  | 0.938 | 0.543 | 2.030 |  |  |  |  |  |  |
| 796.054 |  | -0.060 | -0.022 | 0.505 |  |  |  |  |  |  |
| 796.055 |  | 0.559 | 0.421 | 1.258 |  |  |  |  |  |  |
| 796.055 |  | 8.038 | -0.643 | 4.665 |  |  |  |  |  |  |
| *796.056 |  | -0.083 | 0.725 | 0.729 |  |  |  |  |  |  |
| 796.057 |  | 1.652 | 3.183 | 2.461 |  |  |  |  |  |  |
| 796.057 |  | 0.895 | 1.872 | 3.003 |  |  |  |  |  |  |
| 796.058 |  | 2.505 | 2.424 | 2.017 |  |  |  |  |  |  |
| 796.059 |  | 1.256 | 1.493 | 3.736 |  |  |  |  |  |  |
| 796.059 |  | 0.526 | 2.899 | 2.938 |  |  |  |  |  |  |
| 796.059 |  | 0.445 | -0.277 | 6.156 |  |  |  |  |  |  |
| 796.094 |  | -0.092 | 0.887 | 2.997 |  |  |  |  |  |  |
| 796.060 |  | 1.488 | -0.214 | 3.135 |  |  |  |  |  |  |
| 796.061 |  | 0.132 | 1.964 | 1.183 |  |  |  |  |  |  |
| 796.062 |  | -0.60 | -0.042 | 1.011 |  |  |  |  |  |  |
| 796.069 |  | 0.434 | 0.261 | 1.178 |  |  |  |  |  |  |
| 796.070 |  | 0.447 | -0.195 | 4.104 |  |  |  |  |  |  |
| 796.070 |  | -0.182 | 3.877 | 2.841 |  |  |  |  |  |  |
| 796.071 |  | .263 | 0.439 | 0.318 |  |  |  |  |  |  |
| 796.072 |  | 0.429 | 0.234 | 6.133 |  |  |  |  |  |  |
| *806.102 | 2415.236 | 0.757 | 0.593 | 0.902 | 0.757 | 0.593 | 0.902 | 0.757 | 0.593 | 0.902 |
| *1111.275 | 3330.755 | 1.252 | 1.341 | 7.544 | 1.252 | 1.341 | 7.544 | 1.252 | 1.341 | 7.544 |

h = homology threshold, i = identity threshold, * = peptides above the identity threshold
