# Supplementary material for: A comparison of the accuracy of iTRAQ quantification by nLC-ESI MSMS and nLC-MALDI MSMS methods
Source: J Proteomics. 2010 May 7;73(7):1391–403. doi: 10.1016/j.jprot.2010.03.003 (PMC2880794; doi:10.1016/j.jprot.2010.03.003)
Supplement: Supplementary Table 5 — Effect of applying Mascot% intensity cut-off thresholds to merged triplicate nLC-ESI MSMS data set. [file mmc5.doc]

**Table 5 Supplementary Effect of applying Mascot % intensity cut-off thresholds to merged triplicate nLC-ESI MSMS data set**

**A Aldolase**

| **% int cut-off** | **Mascot**  **threshold** | **No. pep used for quant (115:114, 116:114, 117:114)** | **115:114**  **(exp 2)** | **116:114**  **(exp 4)** | **117:114**  **(exp 8)** | **Mascot score, no. peptides (unique), coverage** |
| --- | --- | --- | --- | --- | --- | --- |
| 0 | Homol  Ident | 179, 179, 185 – 21 uni  69, 71, 71 – 17 uni | 1.46  1.59 | 2.23  2.68 | 3.80  4.84 | 1761, 528 (32),  68% cov |
| 0.5 | Homol  Ident | 162, 170, 173 – 18 uni  74, 77, 77 – 16 uni | 1.54  1.63 | 2.47  2.84 | 4.27  5.05 | 1809, 529 (31), 66% cov |
| 1 | Homol  Ident | 118, 132, 129 – 14 uni  53, 62, 60 – 10 uni | 1.52  1.61 | 2.37  2.78 | 4.06  4.88 | 1857, 528 (31), 66% cov |
| 2 | Homol  Ident | 38, 43, 47 – 2 uni  24, 24, 29 – 2 uni | 1.43  1.65 | 2.01  2.80 | 3.35  4.82 | 1836, 518 (32), 68% cov |

**B Carbonic anhydrase**

| **% int cut-off** | **Mascot**  **threshold** | **No. pep used for quant (115:114, 116:114, 117:114)** | **115:114**  **(exp 0.5)** | **116:114**  **(exp 0.25)** | **117:114**  **(exp 0.125)** | **Mascot score, no. peptides (unique), coverage** |
| --- | --- | --- | --- | --- | --- | --- |
| 0 | Homol  Ident | 270, 269, 262 – 15 uni  183, 181, 180 – 12 uni | 0.60  0.59 | 0.35  0.35 | 0.19  0.20 | 989, 574 (17), 63% cov |
| 0.5 | Homol  Ident | 270, 260, 241 – 15 uni  195, 184, 171 – 13 uni | 0.60  0.59 | 0.35  0.35 | 0.20  0.19 | 1071, 570 (18), 63% cov |
| 1 | Homol  Ident | 230, 209, 190 – 15 uni  171, 155, 140 – 13 uni | 0.60  0.60 | 0.35  0.36 | 0.21  0.21 | 1077, 568 (18), 63% cov |
| 2 | Homol  Ident | 177, 151, 114 – 14 uni  124, 102, 75 – 12 uni | 0.61  0.61 | 0.36  0.36 | 0.24  0.23 | 1067, 561 (18),  63% cov |

exp = expected value

Homol = Homology threshold

Ident = Identity threshold

uni = unique peptides
